# Comprehensive Integration of Single-Cell Transcriptional Profiling Reveals the Heterogeneities of Non-cardiomyocytes in Healthy and Ischemic Hearts

**DOI:** 10.3389/fcvm.2020.615161

**Published:** 2020-12-07

**Authors:** Lingfang Zhuang, Lin Lu, Ruiyan Zhang, Kang Chen, Xiaoxiang Yan

**Affiliations:** ^1^Department of Cardiology, Ruijin Hospital, Shanghai Jiao Tong University School of Medicine, Shanghai, China; ^2^Institute of Cardiovascular Diseases, Shanghai Jiao Tong University School of Medicine, Shanghai, China

**Keywords:** myocardial infarction, single-cell RNA sequencing, immune system, non-cardiomyocytes, macrophages, Ddah1

## Abstract

Advances in single-cell RNA sequencing (scRNA-seq) technology have recently shed light on the molecular mechanisms of the spatial and temporal changes of thousands of cells simultaneously under homeostatic and ischemic conditions. The aim of this study is to investigate whether it is possible to integrate multiple similar scRNA-seq datasets for a more comprehensive understanding of diseases. In this study, we integrated three representative scRNA-seq datasets of 27,349 non-cardiomyocytes isolated at 3 and 7 days after myocardial infarction or sham surgery. In total, seven lineages, including macrophages, fibroblasts, endothelia, and lymphocytes, were identified in this analysis with distinct dynamic and functional properties in healthy and nonhealthy hearts. Myofibroblasts and endothelia were recognized as the central hubs of cellular communication via ligand-receptor interactions. Additionally, we showed that macrophages from different origins exhibited divergent transcriptional signatures, pathways, developmental trajectories, and transcriptional regulons. It was found that myofibroblasts predominantly expand at 7 days after myocardial infarction with pro-reparative characteristics. We identified signature genes of myofibroblasts, such as Postn, Cthrc1, and Ddah1, among which Ddah1 was exclusively expressed on activated fibroblasts and exhibited concordant upregulation in bulk RNA sequencing data and *in vivo* and *in vitro* experiments. Collectively, this compendium of scRNA-seq data provides a valuable entry point for understanding the transcriptional and dynamic changes of non-cardiomyocytes in healthy and nonhealthy hearts by integrating multiple datasets.

## Introduction

Cardiovascular diseases such as myocardial infarction (MI) are a leading cause of morbidity and mortality worldwide. Generally, the difficulties faced while treating ischemia involve not only the poor regeneration of cardiomyocytes after MI, but also the complexity of non-cardiomyocytes (non-CMs), including macrophages, fibroblasts (FBs), and T cells, which play a pivotal role in cardiac remodeling after MI (1). In the last few years, there has been an increased interest in the dynamic changes and transcriptional reprogramming of these cells after ischemia and how these cells coordinate inflammatory and reparative processes during cardiac remodeling (2).

Recently, various studies have been dedicated to the exploration of the dynamic and functional changes of non-CMs after MI using a new method called single-cell RNA sequencing (scRNA-seq) (3), which provides a new entry point for understanding the temporal functions of different cell types in healthy and injured hearts by detecting and analyzing these cells simultaneously (4). These scRNA-seq data have been deposited in public databases, such as the GEO or ArrayExpress database, increasing the possibility of integrating similar scRNA-seq data of non-CMs in healthy and ischemic hearts for a more comprehensive understanding of their dynamics and crosstalk in different states.

In this study, we explored recently published articles on scRNA-seq investigating the functional changes of non-CMs in healthy or ischemic hearts and integrated three representative datasets containing cells collected at 0, 3, and 7 days after MI for a deeper analysis. A total of 27,349 cells, including major non-CM lineages, such as macrophages, FBs, endothelia, and smooth muscle cells (SMCs), were clustered into 20 subpopulations with distinct gene signatures. Analysis of the dynamics and intercellular communication of non-CMs revealed that FBs and endothelial cells (ECs) played a dramatic role in the cellular crosstalk via ligand-receptor interactions. Moreover, the endothelial-like macrophages (MAC-Endo) and FB-like macrophages (MAC-Fib) that exhibited a transcriptional profile of endothelia or FBs under a macrophage background were described in this study. By integrating these scRNA-seq data, we pointed to a novel marker of activated FBs, dimethylarginine dimethylaminohydrolase 1 (Ddah1), which was found to be sharply upregulated after MI in bulk RNA sequencing data and *in vivo* and *in vitro* experiments.

To our knowledge, this is the first study inquiring the possibility of integrating published scRNA-seq data to investigate the transcriptional and dynamic changes of non-CMs in healthy and nonhealthy hearts with unprecedented resolutions.

## Materials and Methods

### Mice and Myocardial Infarction

Male C57BL/6 mice were purchased from the Shanghai Laboratory Animal Center (Shanghai, China) and maintained under a light/dark cycle. All animal experimental procedures were approved by the Animal Care Committee of Shanghai Jiao Tong University School of Medicine. The MI model was used to induced cardiac ischemia *in vivo* as described previously (5). Briefly, 8–10-week-old mice were anesthetized with isoflurane, intubated, and mechanically ventilated with a low concentration of isoflurane gas (1.0%). After opening chest in 3-4 intercostal and removing the pericardium, the left anterior descending artery was ligated with an 8-0 silk suture, followed by closing the chest and skin with 5-0 and 3-0 silk sutures, respectively. In the sham group, all mice underwent an identical procedure except for the artery ligation.

### Analysis and Integration of scRNA-seq Data

The three datasets included in this analysis were downloaded from the GEO database (https://www.ncbi.nlm.nih.gov/geo/) or from ArrayExpress database (https://www.ebi.ac.uk/arrayexpress/) with full expression matrix and mapped Ensemble genes. Firstly, quality control of single cells was performed in every dataset by selecting single viable cells. Specifically, the percentage of mitochondria and the number of features in every cell were set as the limitations. Data on single cells were then filtered according to the source article criteria before the next analysis. After this quality-control procedure, each feature of the cells was normalized by dividing the total unique molecular identifiers and multiplying by 10,000 to obtain a value in transcripts per million, followed by logarithmic transformation using the R package Seurat (6, 7). Notably, we integrated three datasets with a “FindIntegratedanchors” function using 2,000 variable genes differentially expressed among all cells. Then, by conducting “RunPCA,” “FindNeighbors,” and “FindClusters” serially, we were able to partition cell populations according to their expression of marker genes. Notably, the “FindAllMarkers” function in Seurat (with parameters min.pct = 0.25 and logfc.threshold = 0.25) was used to identify specific markers of each cluster, and the results were displayed as a heatmap or uploaded as Supplementary Tables. Then, the “FindMarkers” function was used to identify differentially expressed genes among distinct experimental conditions or among clusters of interest for an enrichment analysis.

### Enrichment Analysis

For pathway enrichment analysis, all cluster-specific markers or differentially expressed genes resulting from the “FindMarkers” function in Seurat were used for Gene Ontology (GO) analysis or Gene Set Enrichment Analysis (GSEA) (8) in RStudio. The R package clusterProfiler (9) was used in this analysis with a cutoff *q*-value of 0.05. The results were generated with ggplot2 as bar plots or GSEA plots and uploaded as Supplementary Tables, and the volcano plots used to compare the expression levels of differential genes between groups were generated using ggplot2 in R.

### Cellular Crosstalk

Cell-cell communication networks were assessed according to a previously published algorithm (10). Briefly, a collection of ligand-receptor pairs generated from the STRING database (11) were further analyzed only if they expressed at least 10% of the cells in a given cell population. Notably, the communication network consisted of source populations expressing differential ligands among clusters and target clusters with corresponding receptors. The weighted path connecting source populations to target clusters via a ligand-receptor pair was calculated as the sum of weights along that path. After adjusting the *p*-value using the Benjamini-Hochberg (BH) method, significant connections between ligands and receptors were included for other analysis at a *p*-value < 0.01. A crosstalk network graph was generated using the R packages circlize (12) and Gephi (https://gephi.org).

### Immunofluorescence (IF) Staining

Immunostaining was performed on the hearts of mice at 0, 3, and 7 days after MI surgery as reported previously (5). After intracardiac perfusion with phosphate-buffered saline and paraformaldehyde, the hearts were fixed for 24 h in 4% paraformaldehyde and subsequently embedded in paraffin and sectioned into 5 μm slices for staining. After antigen retrieval and permeabilization, the slides were blocked with 5% bovine serum albumin buffer for 30 min and then probed overnight with primary antibodies. After washing with phosphate-buffered saline and incubation with the corresponding Alexa Fluor-conjugated secondary antibodies for 2 h and 4′,6-diamidino-2-phenylindole for 5 min. Images were taken using a fluorescence microscope. The following antibodies were used for IF staining: Ddah1 (ab180599, 1:100; Abcam, Cambridge, UK) and α-smooth muscle actin (sc-53142, 1:100; Santa Cruz Biotechnology, Inc., Dallas, TX, USA). In all quantifications of immunofluorescence images, we performed at least three individual samples for every group and acquired at least five high-resolution images for every section.

### Western Blot

Total protein was extracted from the heart tissues of mice after MI or sham surgery using a sodium dodecyl sulfate lysis buffer, and then protein concentrations were measured using a BCA assay kit. Western blot analysis was performed as described previously (5). Briefly, protein samples were separated using sodium dodecyl sulfate-polyacrylamide gel electrophoresis, transferred to a polyvinylidene fluoride membrane (Millipore, Burlington, MA, USA), and incubated with 5% fat-free milk for 1 h before being incubated with primary antibodies at 4°C overnight. Antibody Ddah1 (ab180599, 1:1,000; Abcam) was used, and GAPDH was used as the control.

### Pseudotime Trajectory Analysis

Pseudotime analysis was specifically performed on macrophage and FB lineages from our integrated single-cell data using the R Package Monocle (13). The unique expression matrix and cell identities were fetched from Seurat files and used as input material for further analyses. After integrating and reducing dimensions with default parameters, clusters were defined using the “cluster_cells” function with a resolution of 10^−3.3^, and their pseudotime trajectory was determined using the “learn_graph” function. The results were plotted in three-dimensional space. To analyze differentially expressed genes triggering a particular pseudotime distribution of cell populations, we used the “graph_test” function to investigate differential genes among cell clusters.

### Transcriptional Regulon Analysis

Core regulatory transcription factors of macrophages and T cell lineages were predicted using the R package SCENIC (14). The GENIE3 package (Step 1) was used to infer gene regulatory networks from the expression matrix of the included cells on the basis of the coexpression networks. Then, a RcisTarget analysis (Step 2) was performed to investigate the DNA motif and predict regulons among cell populations on the basis of the “mm9-tss-centered-10kb” database. The activity of the regulatory networks was evaluated on the full dataset in the scoring step with AUCell (Step 3). Notably, regulons annotated as “extended” included target genes harboring motifs that have been linked to the respective transcription factor by lower confidence annotations.

### Statistical Analysis

Data were presented as box-and-whisker plots or bar plots with all points, which were evaluated and organized using the commercial software GraphPad Prism (version 7.0a; GraphPad Software, San Diego, CA, USA). Statistically significant differences between every two groups were analyzed using Student's two-sided *t*-test. For experiments with more than two groups, after confirming normality and homogeneity of variance, one-way analysis of variance (ANOVA) followed by Tukey's post hoc test was used for comparison. For comparison between multiple experimental groups with a control group, the Dunnett's post hoc test was used in SPSS Statistics (version 23; IBM, Armonk, NY, USA). For all analyses, statistical significance was set at *p* < 0.05.

## Results

### Integration of the Three Single-Cell RNA Sequencing Datasets Exhibits a Cell-Type-Dependent Distribution of Non-cardiomyocytes

**Figure 1:**
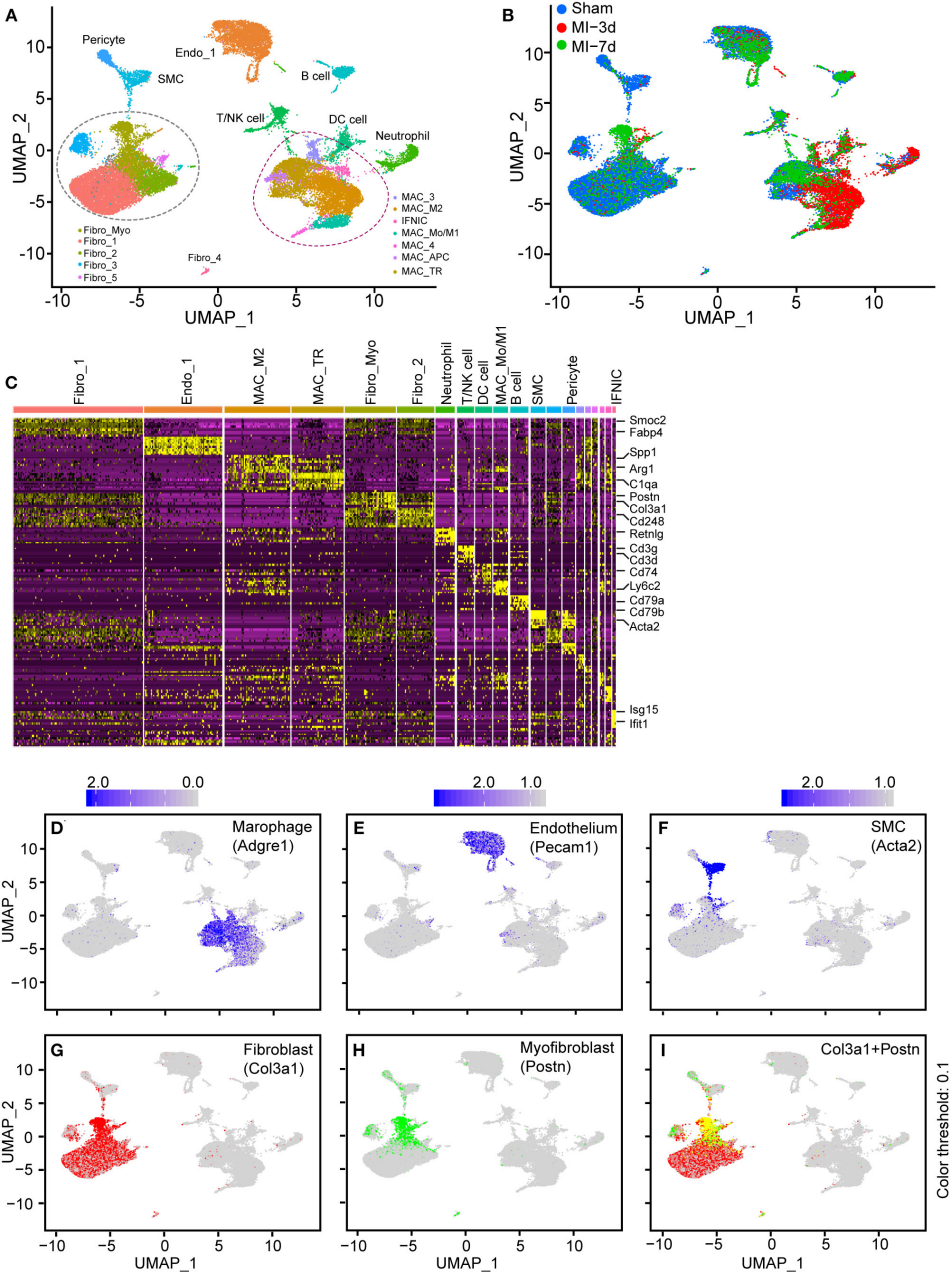
Integration of non-cardiomyocytes (non-CMs) in healthy and nonhealthy hearts. **(A)** UMAP plot of non-CMs from the sham group or 3 and 7 days after myocardial infarction (MI), showing the construction of 20 subpopulations in different colors. **(B)** UMAP plot of integrated non-CMs under different conditions shown in different colors. **(C)** Heatmap of 20 subclusters with differentially expressed genes across cell populations for cell identification, in which the upper bars refer to subclusters. **(D–I)** UMAP visualization of all cells, combined with the expression of Adgre1 **(D)**, Pecam1 **(E)**, Acta2 **(F)**, Col3a1 **(G)**, and Postn **(H,I)**. The scaled expression of target genes were indicated in the upper portion of each panel.

First, to investigate the possibility of integrating similar scRNA-seq data, we collected and summarized recently published single-cell sequencing data of mouse hearts (Supplementary Table I). Three articles were chosen for further analysis (10, 15, 16): (1) isolating and sequencing CD45+ leukocytes from MI-operated hearts at 4 days after surgery (*n* = 1,866) or sham-operated hearts (*n* = 703) (15) (Supplementary Figures 1A,B); (2) isolating all interstitial cells from murine hearts at 3 days (*n* = 4,067), and 7 days (*n* = 4,194) after MI or sham (*n* = 5,977) surgery (10) (Supplementary Figures 1C,D); (3) collecting nonmyocyte cells from the ventricles of mouse hearts (16) under a homeostatic condition (*n* = 10,542; Supplementary Figure 1E). After a similar quality-control procedure according to their source study, each independent dataset was processed with an unsupervised clustering algorithm to identify cell lineages and visualize with t-SNE plots (6) (Supplementary Figure 1). Next, to find the correspondences among these datasets, we harmonized these datasets into a single reference using the “FindIntegratedanchors” function in Seurat (7) and visualized the distributions of cells with UMAP reduction plots. A total of 27,349 non-CM cells constituting nine cell lineages were partitioned into 20 subpopulations depending on their dominant expressed marker genes (Figure 1A). Of note, each cluster consisted of cells from all conditions without showing significant batch effects, indicating the reliability of our integration strategy (Figure 1B).

In this analysis, cells were clustered and designated depending on the concordant expression of well-characterized marker genes (Supplementary Table II, Figure 1C). Specifically, secreted phosphoprotein 1 (Spp1) (17) and arginase 1 (Arg1) (18, 19), which are dramatically upregulated on M2 macrophages, were found to be predominantly expressed in the MAC-M2 cluster, whereas periostin (Postn) (20) was found to exclusively exist in activated FBs (Fibro_Myo; Figure 1C). Additionally, T cells/natural killer (NK) cells (expressing Cd3g and Cd3d), B cells (expressing Cd79a and Cd79b), and SMCs (expressing Acta2) were also identified in this study (Figure 1C). Furthermore, the UMAP plots of curated conservative marker genes indicated well-partitioned cell lineages. Specifically, the cell-type-dependent expression of Adgre1 (marker of macrophages; Figure 1D), Pecam1 (marker of ECs; Figure 1E), Acta2 (marker of SMCs; Figure 1F), Col3a1 (marker of FBs; Figure 1G), Postn (marker of myofibroblasts; Figures 1H,I), Cd79a (marker of B cells; Supplementary Figure 1F), Cd8b1 (marker of T cells; Supplementary Figure 1G), and Vtn (marker of pericytes; Supplementary Figure 1H) confirmed the well-clustered cell populations in our integrated scRNA-seq data, providing us with an important basis to further explore the dynamics and functional changes of each cell cluster.

Taken together, these findings demonstrate that, by integrating different datasets, we were able to analyze non-CMs in healthy and injured hearts in a single-cell resolution.

### Cell Dynamics and Cellular Communication Analysis Reveal Temporal and Functional Differences Between Cell Types

**Figure 2:**
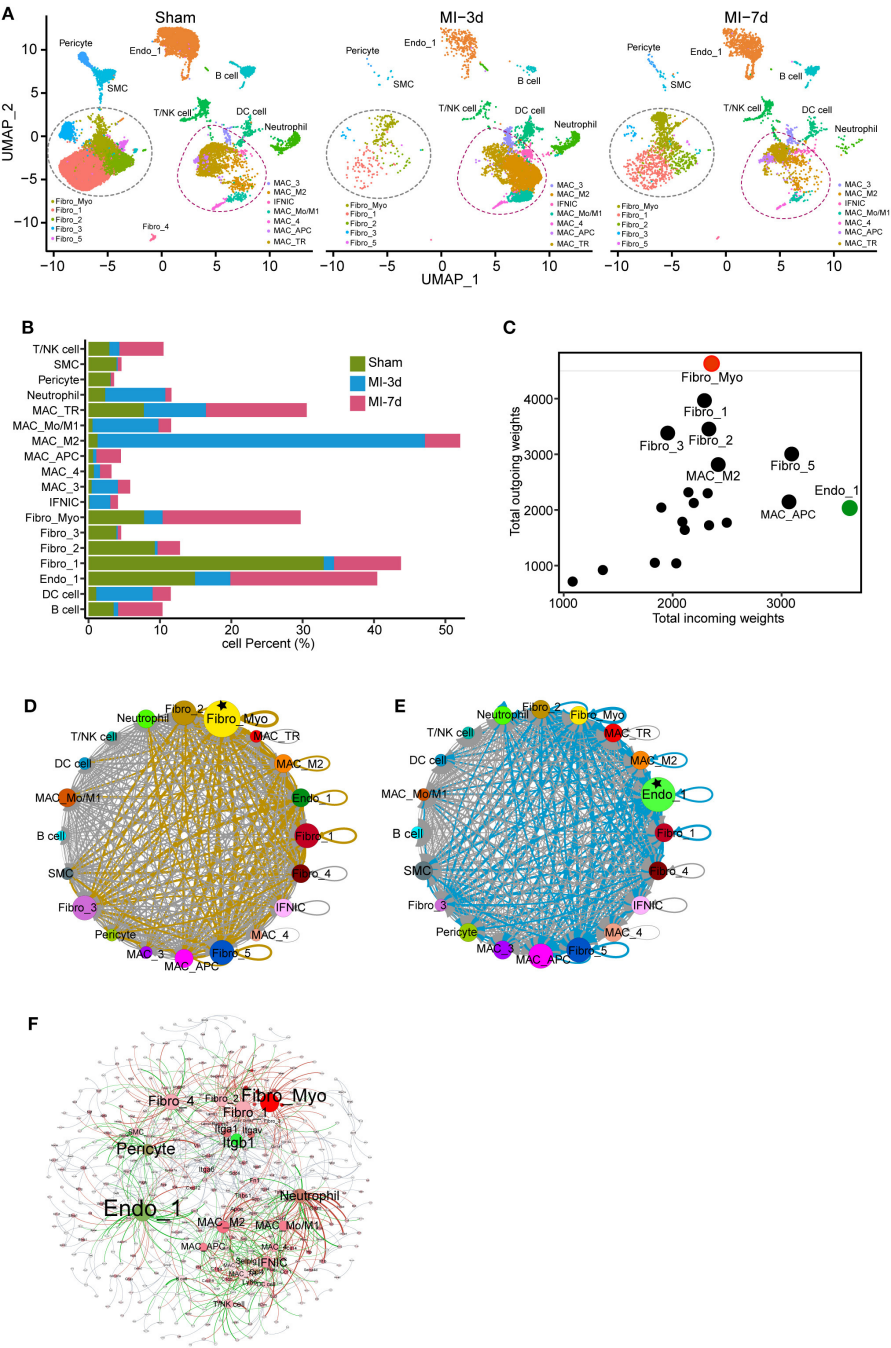
Dynamics and cell communication of non-cardiomyocytes (non-CMs). **(A)** Separated UMAP plots showing heterogeneous non-CM populations in the sham group, 3 and 7 days after myocardial infarction (MI). Fibroblasts (FBs) and macrophages were circled with dashed lines. **(B)** Bar plot showing the dynamics of cell clusters in sham and MI hearts. **(C)** Cell communication across all subpopulations was identified with well-characterized ligand-receptor pairs. Outcoming and incoming weights refer to the weighted numbers of ligands and receptors among all cell populations. **(D,E)** Putative cell communication based on differentially expressed ligands **(D)** and receptors **(E)** including total cell clusters. Circular compartments representing subclusters with weighted sizes. **(F)** Putative cell-cell interactions, including cell types, ligands, and receptors. All interactions of ligands from source cell populations to receptors (target populations) were plotted; cell type, ligands, and receptors were filled as nodes; and interactions were shown as edges. The source data were uploaded as Supplementary Table III.

To delineate the composition of each cluster reflecting disease-related cell fluxes under ischemic conditions, separated UMAP plots including cells from sham, 3 or 7 days after MI (MI-3d, MI-7d, respectively) were mapped for a more directed visualization of cell composition during healing process (Figure 2A, Supplementary Figure 2A). We observed drastically ischemia-induced cell fluxes. For example, the expansion of the MAC_M2 population peaked at 3 days after MI surgery and the proportion of Fibro_Myo sharply increased at 7 days after MI, as previously reported (21). Besides, significantly infiltrated T/NK and B cells were observed at 7 days after MI, whereas neutrophil and dendritic cell populations dramatically expanded at 3 days (Figure 2B). This distinct time window of cell expansion after MI suggests varied functions of non-CMs during cardiac repair and remodeling process.

Increased cell-cell communication is a hallmark of cardiac repair and plays a pivotal role in cardiac remodeling (22). To investigate the crosstalk of non-CMs, we analyzed the expression of ligands and receptors in each cell population using a curated collection of human ligand-receptor pairs (23) and the STRING database (11). Then, after computing their weighted expression among source and target populations (cells with differentially expressed ligands referred to source clusters and cells with upregulated receptors related to target populations), a total of 636 ligand-receptor pairs were identified in our data with differential expression on total cell populations (Supplementary Table III). By summarizing all the weighted paths, we found that FBs possess the largest number of ligands among all cell clusters. More specifically, the Fibro_Myo, Fibro_1, Fibro_2, Fibro_3, and Fibro_5 populations were recognized as the top five clusters with dominant outbound connections. Moreover, Fibro_Myo have the most prominent number of differentially expressed ligands, suggesting their indispensable function in communicating with other cells for cardiac repair (Figures 2C,D). Besides, it is intriguing that ECs (Endo_1) were found to have the most weighted path as the target population, implying their crucial role in ligand binding and cell response (Figures 2C,E) after MI. An integrated map including cell clusters, ligands, and receptors (Figure 2F) confirmed our previous analysis, underscoring the centrality hub roles of Fibro_Myo and Endo_1 as source and target populations in cell-cell communication. Notably, we found that the integrin family, including integrin subunit beta 1 (Itgb1), had the largest weighted connections with ligands expressed in multiple cell populations (Figure 2F). Collectively, these observations emphasize the profound function of FBs and ECs in the repair process.

### Macrophage/Monocyte Populations in Healthy and Injured Hearts

**Figure 3:**
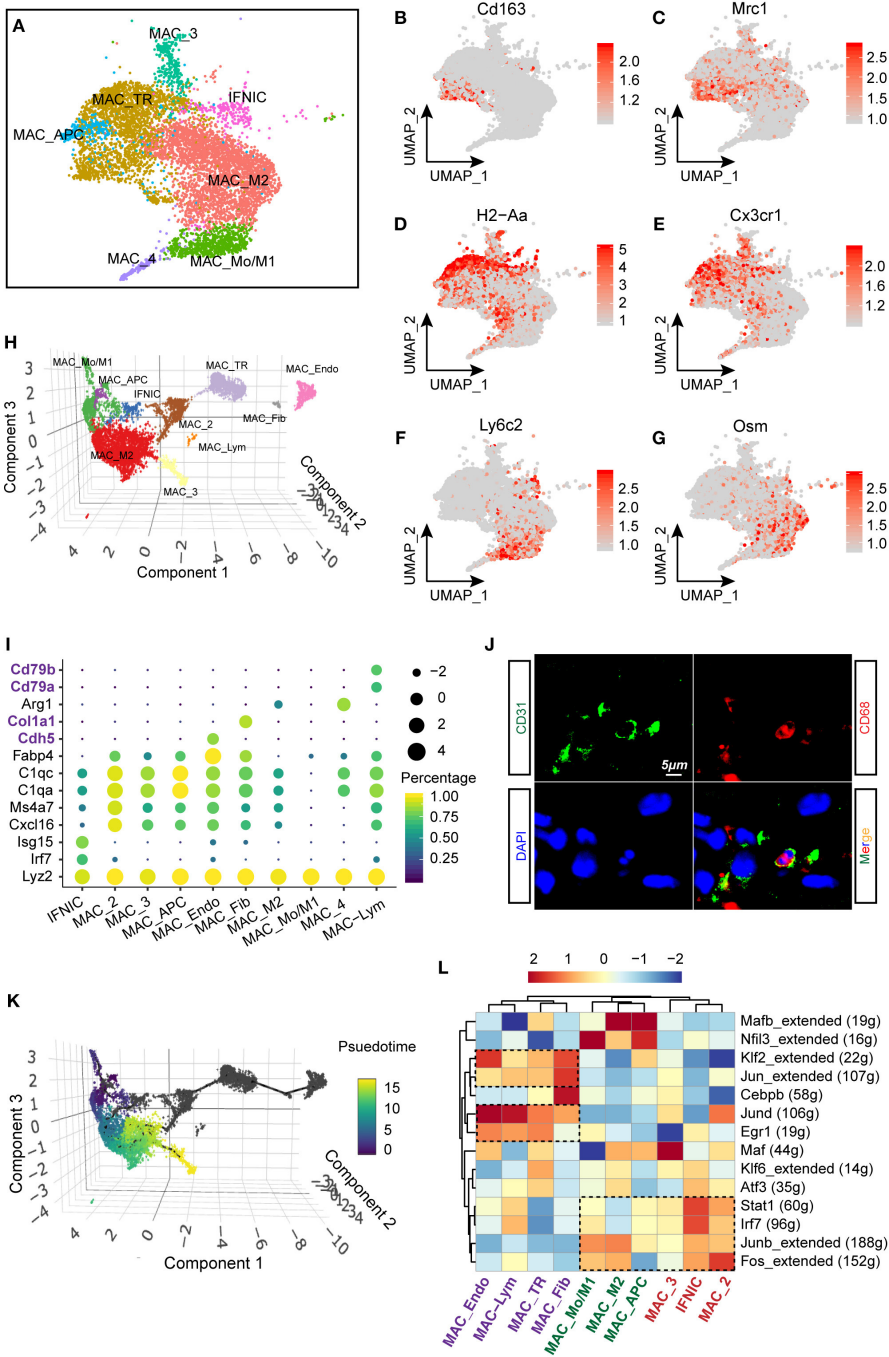
Heterogenous gene signatures, transcriptional regulons of cardiac macrophages. **(A)** UMAP plot with all macrophage subclusters were visualized with different colors corresponding to their identity. **(B–G)** Gene expression of Cd163 **(B)**, Mrc1 **(C)**, H2-Aa **(D)**, Cx3cr1 **(E)**, Ly6c2 **(F)**, and Osm **(G)** within macrophage lineages were visualized with UMAP plots. The right bar indicated the scaled expression of each gene in macrophage subpopulations. **(H)** Reconstruction of macrophage clusters in Monocle showing the heterogeneity of cell populations. **(I)** Dot plot showing the expression of cell-type-specific signatures in identified cell clusters in Monocle. The dot size and color represented the mean expression and proportion of each cell population expressing genes, respectively. **(J)** Immunofluorescent costaining of CD31 and CD68 in mouse heart sections. **(K)** Developmental trajectory analysis including all macrophage clusters showing the pseudotemporal development of included cells. On the right, the scale going from dark to light color represented the process of maturity. **(L)** Single-cell regulatory network inference and clustering (SCENIC) analysis of macrophage clusters to investigate differentially regulated transcriptional factors, were showed as a heatmap. The dashed rectangles indicated enriched regulons among specific clusters.

As macrophages constitute the largest number of immune cells in the heart and exhibit a dramatic cell flux in injured hearts (Figures 2A,B), it was therefore important to figure out not only the flux of macrophages, but also its functional differences under homeostatic and ischemic conditions. In this study, a total of seven subclusters of macrophages were identified (Figure 1A) and selected for deeper analysis (specifically IFNIC, MAC_Mo/M1, MAC_M2, MAC_TR, MAC_APC, MAC_4, and MAC_3; Figure 3A). In line with a previous study (24), we observed distinct tissue-resident (TR) macrophages (MAC_TR) that exhibited increased expression of Cx3cr1 and H2-Aa (Figures 3D,E) and downregulated expression of the proinflammatory gene Ly6c2. Among these TR macrophages, some cells displayed exclusively expressed proreparative genes, Cd163 and Mrc1 (Figures 3B,C), while lacking H2-Aa compared to others, suggesting that these cells assume more anti-inflammatory roles during ischemic injuries rather than antigen processing and presentation, consistent with other single-cell sequencing research on cardiac hypertrophy models (25). Besides, some proinflammatory macrophage populations with higher levels of Ly6c2 and oncostatin M (Osm; Figures 3F,G) were found to be far from TR macrophages, suggesting dramatic differences in cell phenotypes and molecular functions between these two types of cell populations.

Furthermore, we aligned all macrophage populations for pseudotemporal analysis to reconstruct the possible trajectory of macrophages and determine the regulators of cell fate using the R package Monocle (13, 26). A three-dimensional UMAP plot revealed 10 separate cell clusters, including MAC_Mo/M1, MAC_M2, IFNIC, and MAC_TR, which have been verified in the Seurat algorithm. Additionally, we observed the existence of FB-like (MAC_Fib) and endothelial-like (MAC_Endo) macrophages in our analysis (Figure 3H). These cell populations showed exclusive expression of FB markers (Col1a1) and endothelial markers (Cdh5) in macrophages (Figure 3I). In fact, several previous studies confirmed the possibility that macrophages would transform into FB-like cells during MI remodeling (27); however, the possibility of whether macrophages can transform into endothelia still requires further exploration. In line with the scRNA-seq results, we validated the existence of endothelial-like macrophages by costaining CD31 and CD68 using immunofluorescence technology (Figure 3J). Additionally, by ordering the pseudotime cell trajectory, we described a continuous stage of macrophages starting from early infiltrating monocytes and M1 macrophages to late proreparative M2 macrophages (Figure 3K), which is similar to what has been found in previous studies on single-cell analysis (10) or *in vivo* experiments (21). However, it was found that other macrophage subpopulations, such as MAC_TR, MAC_Fib, and MAC_Endo, are hard to merge into the trajectory of blood-derived macrophages, suggesting a divergent origin and transcriptional profile of these two cell types.

Next, we performed single-cell regulatory network inference and clustering (SCENIC) analysis (14) to investigate the core transcriptional regulons driving the differentiation of distinct macrophage subpopulations. Intriguingly, we discovered that blood-derived macrophages, such as MAC_Mo/M1 and IFNIC, exhibit activated proinflammatory regulons, for instance, signal transducer and activator of transcription 1 (Stat1) and interferon regulatory factor 7 (Irf7). In line with previous researches, it was found that excessive Irf3 activation and interferon production trigger the overproduction of cytokines and infiltration of immune cells after MI (15). Besides, early growth response 1 (Egr1) and Jund regulons were predominantly enriched in TR macrophages, such as MAC_TR, MAC_Fib, and MAC_Endo. Corroborating our findings, previous studies confirmed that the zinc finger transcription factor Egr1 is essential for the activation and differentiation of macrophages (Figure 3L) (28, 29). Taken together, these data provide new insights into the heterogenous transcriptional profile and regulons of macrophages.

### Transcriptional Differences of Tissue-Resident and Blood-Derived Macrophages

**Figure 4:**
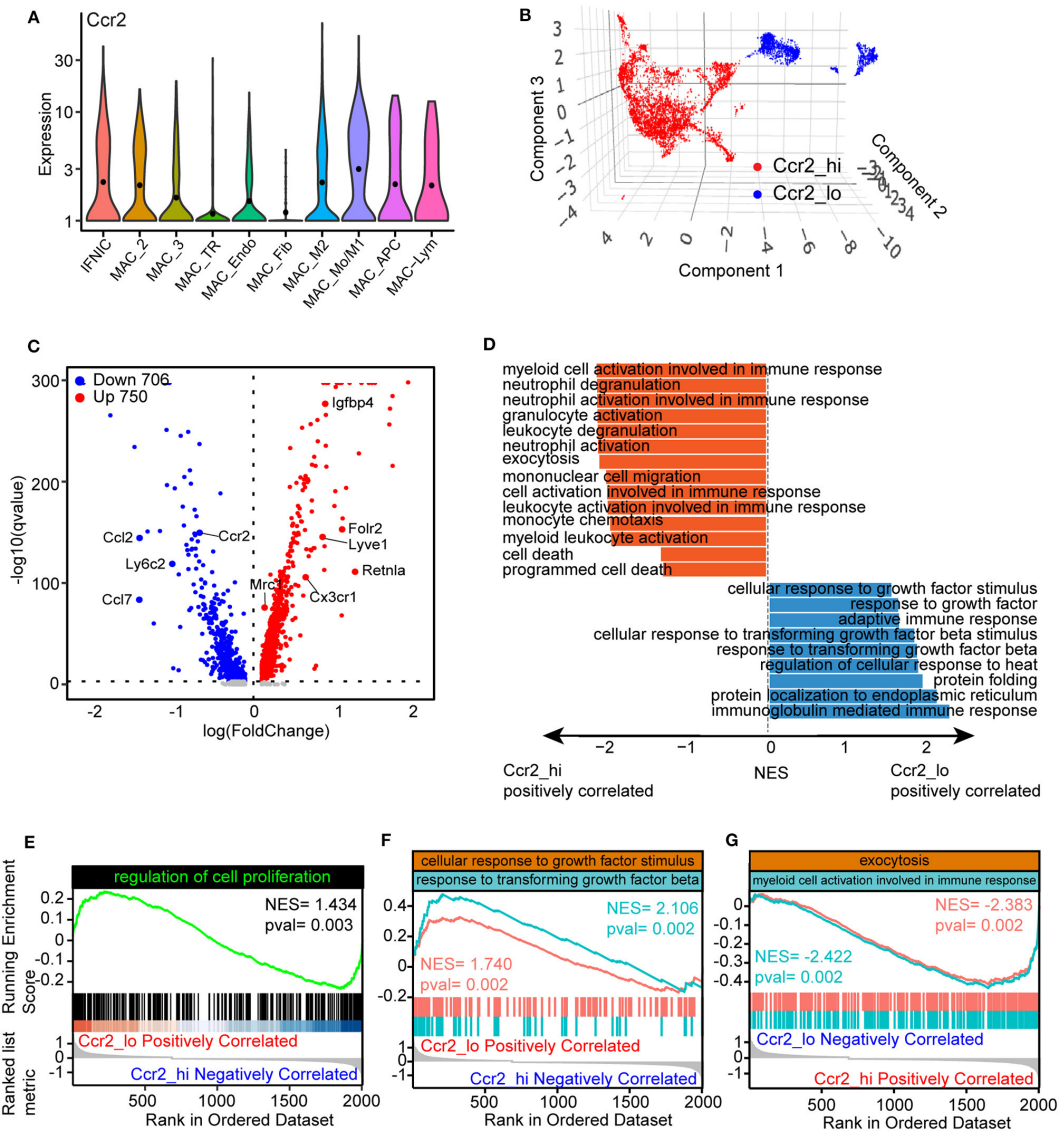
Divergent function of tissue-resident (TR) and blood-derived macrophages. **(A)** Violin plots showing the expression of Ccr2 in macrophage clusters. **(B)** 3D UMAP plot with reassigned cell identities according to the level of Ccr2 in the macrophage populations. Specifically, MAC_TR, MAC_Endo, and MAC_Fib were renamed as Ccr2_lo, whereas others were denoted as Ccr2_hi. **(C)** Volcano plots comparing differentially expressed genes between Ccr2_lo and Ccr2_hi clusters, with each dot representing one gene. **(D)** Gene set enrichment analysis (GSEA) of differentially expressed genes between Ccr2_lo and Ccr2_hi clusters using ClusterProfiler. The results were displayed as normalized enrichment score (NES) between Ccr2_lo and Ccr2_hi group. **(E–G)** Visualization of gene sets including the regulation of cell proliferation **(E)**, cellular responses to growth factor stimulation and response to transforming growth factor-β (TGF-β) **(F)**, and exocytosis and myeloid cell activation involved in immune responses **(G)** comparing Ccr2_lo macrophages with Ccr2_hi clusters.

In our previous work, we observed that macrophages with varied origin exhibit divergent developmental trajectory and distinct transcriptional regulons. To explore the differences of gene signatures and pathways among TR and blood-derived macrophages, we compared the expression patterns of Ccr2, a well-known marker that distinguishes TR macrophages from blood-derived macrophages. Markedly downregulated expression of Ccr2 was observed in MAC-TR, MAC_Endo, and MAC_Fib clusters, compared to other cell populations (Figure 4A). Next, to more specifically determine the underlying expression patterns of TR macrophages, we reassigned the three aforementioned cell clusters as a Ccr2-low cluster (Ccr2_lo) and the others as a Ccr2-high cluster (Ccr2_hi) and mapped using a UMAP plot (Figure 4B). By comparing differentially expressed genes among these two groups, Ccr2_lo cells showed lower expression of Ccr2 and Ly6c2, whereas the marker gene of proreparative macrophages Mrc1 was upregulated. Moreover, lymphatic vessel endothelial hyaluronan receptor 1 (Lyve1) and Cx3cr1, which have been recognized as markers of TR macrophages, were found to be highly expressed in the Ccr2_lo group (Figure 4C) (30). We also performed GSEA (8) on the basis of biological process terms and observed prominent differences in the activated pathway patterns between these two groups (Figure 4D, Supplementary Table IV). Specifically, the Ccr2_lo group was found to exhibit significantly enriched gene sets involved in the regulation of cell proliferation (Figure 4E) and cellular response to growth factors or transforming growth factor beta (TGF-β) (Figure 4F), in agreement with the activation of Egr1 transcriptional regulons in TR macrophages. However, it was found that myeloid cell activation, which is involved in immune responses and exocytosis, two key pathways associated with proinjury macrophages, is positively correlated with Ccr2_hi macrophages (Figure 4G). Overall, functional comparative analysis of Ccr2_lo and Ccr2_hi macrophages supported the protective role of TR macrophages by exerting self-renewing and anti-inflammatory effects after MI.

### Flux of Fibroblasts and Activated Fibroblasts in Ischemic Hearts

**Figure 5:**
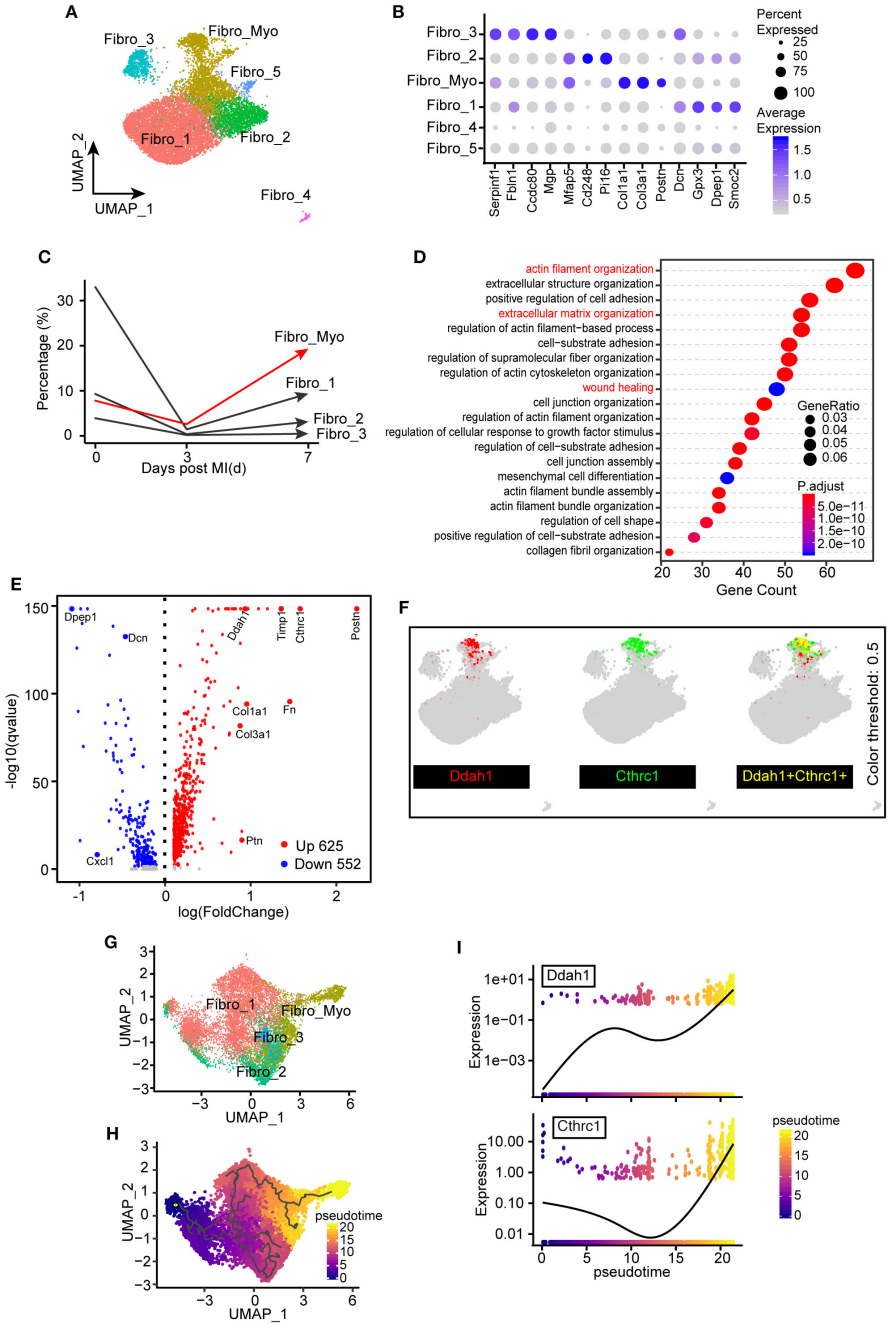
Identification of the distinct transcriptional diversity of myofibroblasts in ischemic responses. **(A)** Subclustering of fibroblast (FB) populations for the next analysis. UMAP plot including all cell clusters with different colors. **(B)** Dot plot of selected marker genes corresponding to their cell identity. The dot size and scale colored represented the percentage of expressed genes and the mean expression of each cell population, respectively. **(C)** Percentages of different FB clusters (Fibro_Myo, Fibro_1, Fibro_2, and Fibro_3) at 0, 3, and 7 days after myocardial infarction (MI) surgery. **(D)** Hallmark gene ontology (GO) analysis between myofibroblasts (Fibro_Myo) and all other clusters based on biological process terms. **(E)** Volcano plot of differentially expressed genes between myofibroblasts and others clusters. The red dots indicated upregulated genes, whereas the blue dots indicated decreased genes. **(F)** UMAP plots showing the coexpression of myofibroblast marker genes (Ddah1, Cthrc1) in all FB populations. **(G)** Repartitioning of all FBs based on the Monocle algorithm. **(H)** Pseudotime trajectory analysis of all FBs according to highly variable genes. UMAP plot showing pseudotime score from dark to light yellow, representing the early to terminal transition. **(I)** Pseudotime expression of Ddah1 and Cthrc1, indicating their continuously upregulated expression during the healing process.

As FBs constitute the largest number of non-CMs under homeostatic conditions and play an indispensable role in fibrotic scar formation and cardiac remodeling after MI, our next aim was to explore the functional changes of FBs in healthy and MI-operated hearts. In total, six FB clusters were identified (Figures 1A, 5A) in this study: Fibro_1, Fibro_2, Fibro_3, Fibro_4, Fibro_5, and Fibro_Myo. Comparison of marker genes among these clusters indicated that myofibroblast markers (31), such as Col1a1, Col3a1, and Postn, are significantly upregulated in Fibro_Myo clusters (Figure 5B). Further exploring the flux of FBs after MI, we found that, compared with the sham surgery, the proportions of FBs were reduced at 3 days after MI, which may have resulted from a sharp ischemic condition and loss of FBs. Notably, myofibroblasts (Fibro_Myo) prominently increased at 7 days after MI compared with other FB clusters, suggesting an important role of Fibro_Myo in ischemic responses and healing processes (Figure 5C). Additionally, GO analysis of Fibro_Myo with other FB clusters showed significant enrichment of prohealing pathways, such as actin filament organization, extracellular matrix (ECM) organization, and wound healing (Figure 5D).

We then compared differentially expressed genes between Fibro_Myo and five other clusters and observed dramatic upregulation of Cthrc1, which has been confirmed to be a marker of myofibroblasts (32), and Ddah1 (Figure 5E). Some previous studies confirmed that Ddah1 plays a crucial role in the clearance of asymmetric dimethylarginine and monomethyl arginine, which are strongly associated with premature cardiovascular disease and death (33). By combining the expression of Cthrc1 and Ddah1, we found exclusively co-expressed Cthrc1 and Ddah1 in myofibroblasts rather than in other FB clusters (Figure 5F), suggesting that Ddah1 may serve as another marker of myofibroblasts.

Next, to investigate the trajectory development of FBs and myofibroblasts, we reclustered the FB populations in the Monocle package for pseudotime analysis (Figure 5G). Intriguingly, we found a continuous cell development from Fibro_1 to activated FBs (Fibro_Myo), consistent with their dynamic changes after MI (Figure 5H). As myofibroblasts exist in the final stage of cell trajectory, we next analyzed a pattern of gene expression that triggers this developmental trajectory using the “graph autocorrelation analysis” function embedded in Monocle. Unexpectedly, we found that Cthrc1 and Ddah1 are positively correlated with the development of myofibroblasts, suggesting their crucial effect on the activation of FBs (Figure 5I). Taken together, these observations indicate that the heterogeneities and dynamic changes of FBs during healing process and Ddah1 exclusively expressed in myofibroblasts may represent another marker of activated FBs.

### Verifying the Expression and Location of Ddah1 After Myocardial Infarction

**Figure 6:**
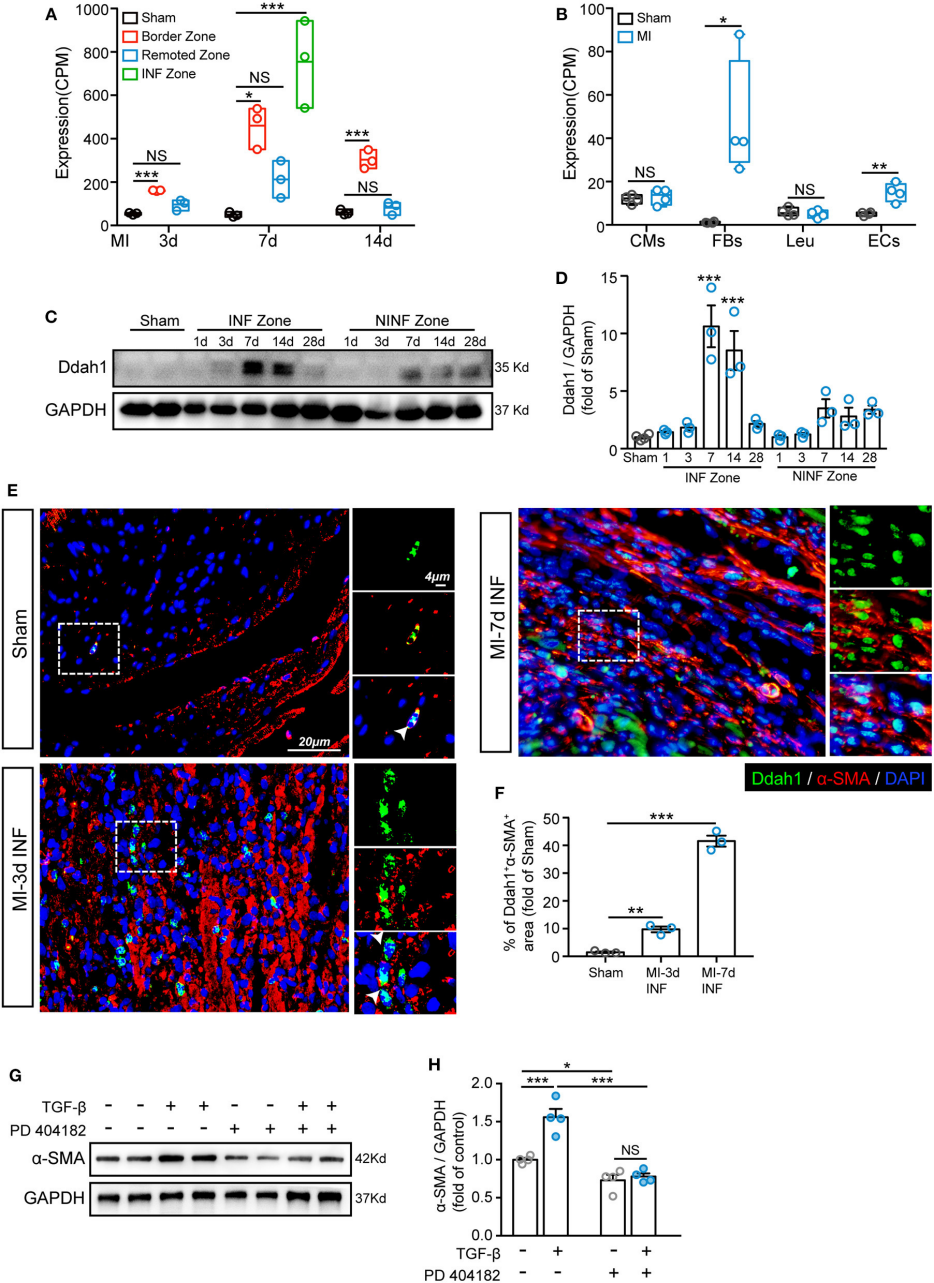
Upregulation of Ddah1 after myocardial infarction (MI). **(A)** Recalculation the expression of Ddah1 in bulk RNA sequencing data from border, remoted, and infarcted (INF) zones at 3, 7, and 14 days after MI or sham surgery. **(B)** Recalculation the expression of Ddah1 in bulk RNA sequencing data from cardiomyocytes (CMs), fibroblasts (FBs), leukocytes (Leu), and endothelial cells (ECs) at 3 days after MI or sham surgery. **(C)** Immunostaining of Ddah1 in the INF zone and non-INF (NINF) zone from MI-operated hearts at 0, 1, 3, 7, 14, and 28 days. GAPDH was used as the control. **(D)** The quantification results of C. The data were expressed as the mean ± SEM. **(E)** Immunofluorescence co-staining of Ddah1 (green) and α-smooth muscle actin (α-SMA, red) in the sham group and the infarcted zone (INF) at 3 and 7 days after MI surgery. Nuclei were stained using 4′,6-diamidino-2-phenylindole (DAPI, blue). On the right, high-magnification images were shown, corresponding to the dashed rectangle in the images on the left. **(F)** The quantification results of panel **E**. **(G)** Western blot analysis showed the expression of α-SMA in fibroblasts in response to TGF-β or Ddah1 inhibitor, PD 404182. GAPDH was used as the control. **(H)** The quantification results of G. The data were expressed as the mean ± SEM, NS, not significant; *p < 0.05, **p < 0.01, ***p < 0.001. (**A**, *n* = 3, Tukey post hoc test; **B**, *n* = 4, Student's *t* test; **D,F**, *n* = 3, Dunnett *post hoc* test; **H**, *n* = 4, Tukey post hoc test).

It has been confirmed in a previous analysis that Ddah1 has a distinct function in the development and activation of FBs (Figure 5). To investigate and verify the expression of Ddah1 in ischemic hearts, we first recalculated the expression of Ddah1 in two curated articles in which bulk RNA sequencing was performed: (1) Transcriptome analysis of remote, border, and infarcted zones in mice at 3, 7, and 14 days after MI or sham surgery (PMID: 31259610) (34); (2) Transcriptome analysis of cardiomyocytes, FBs, leukocytes, and ECs from sham- or MI-operated neonatal (P1) and adult (8W) mouse hearts at 3 days after surgery (PMID: 28733351) (35). From the first dataset, we discovered that ischemia induced robust upregulation of Ddah1 in the border zone at 3, 7, and 14 days after surgery, whereas its expression peaked at 7 days in the infarcted zones (Figure 6A). Moreover, the second article, which analyzed the transcriptional expression of four cell types, indicated that upregulated Ddah1 after MI surgery exclusively existed in FBs and ECs, while lacking any significance in CMs and leukocytes (Leu; Figure 6B). Consistent with the first RNA-seq results, immunoblotting of Ddah1 in the sham group and at 1, 3, 7, 14, and 28 days after MI showed a drastic increase of Ddah1 in the infarcted zone at 7 and 14 days after MI operations (Figures 6C,D, Supplementary Figure 5A).

Next, double immunofluorescence staining of Ddah1 and fibroblasts marker, vimentin, was conducted to evaluate the expression and location of Ddah1 *in vivo* and *in vitro*. Consistent with boosted Ddah1 protein level at 7-days after MI, we showed increased Ddah1^+^vimentin^+^ cells at 7-days after MI surgery compared with sham hearts (Supplementary Figures 3A,B). Furthermore, fibroblasts were isolated from neonatal rat hearts and treated with or without TGF-β before immunofluorescence analysis, the results revealed that TGF-β stimulation dramatically upregulated Ddah1 protein level in fibroblasts *in vitro* (Supplementary Figures 3C,D).

Next, we examined the functional role of Ddah1 in the activation of fibroblasts into myofibroblasts. First, isolated cardiac fibroblasts were treated with TGF-β and co-stained with Ddah1 and α-SMA, the results revealed that activated fibroblasts expressed more Ddah1 in response to TGF-β stimulation *in vitro* (Supplementary Figures 3E,F). Consistently, increased Ddah1^+^α-SMA^+^ cells were identified in the infarcted (INF) and border zones at 3-days after MI operations vs. sham group, and these cells were significantly expanded at 7-days post-surgery (Figures 6E,F, Supplementary Figure 4A). Finally, isolated cardiac fibroblasts were treated with TGF-β or Ddah1 inhibitors (PD 404182) to explore its role on activation of fibroblasts. The myofibroblasts activation, as evaluated by α-SMA expression, was strongly increased in response to TGF-β stimulation and reduced when Ddah1 was inhibited by PD 404182 (Figures 6G,H, Supplementary Figure 5B). Thus, these findings suggest that Ddah1 is upregulated in FBs after MI stimulation and may participate in cardiac remodeling after MI by modulating the activation of myofibroblasts.

### Gene Reprogramming and Dynamic Changes of Endothelial and T Cells

**Figure 7:**
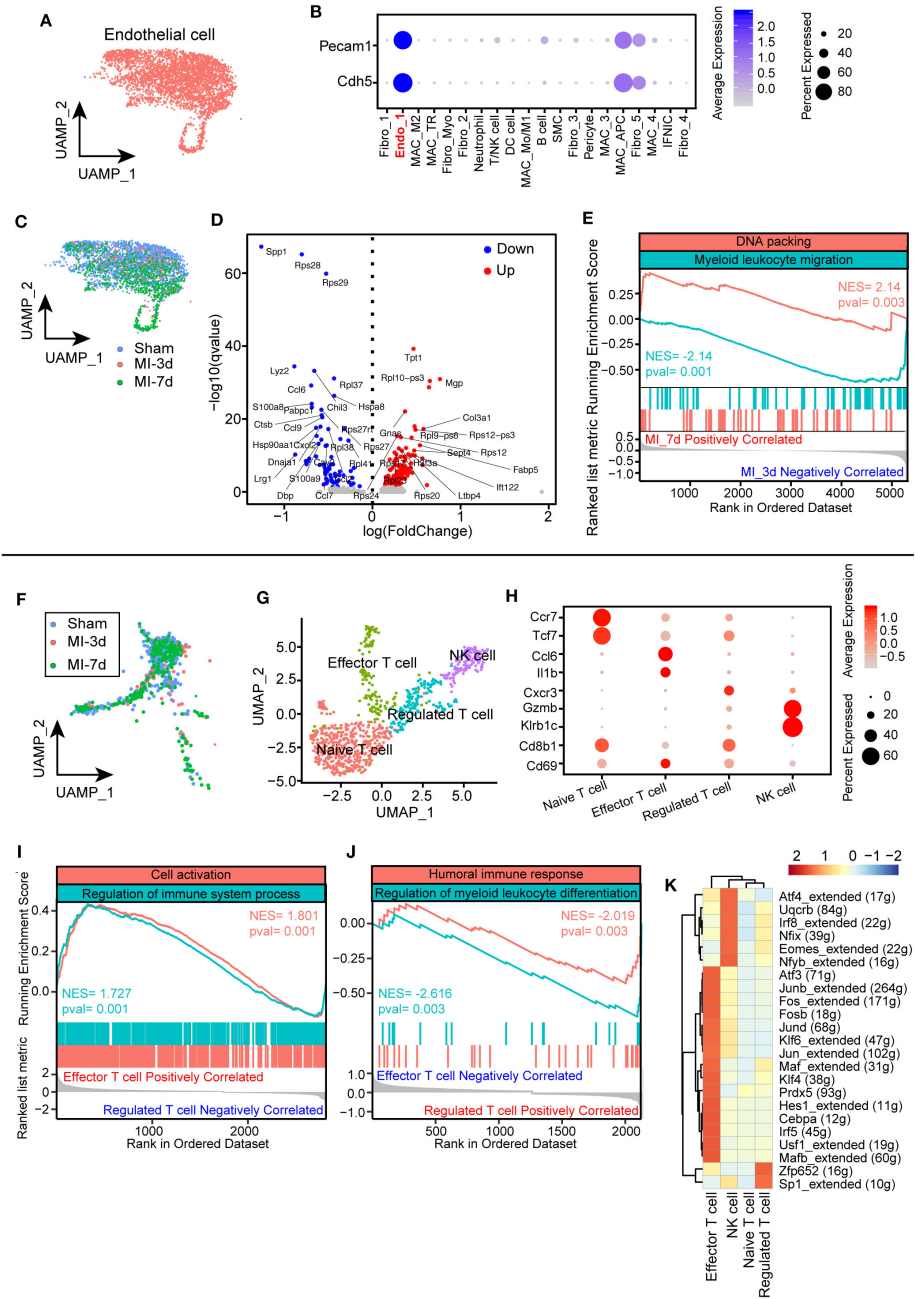
Characterization of endothelial cells (ECs) and T cells after myocardial infarction (MI). **(A)** UMAP plots of ECs in this study selected from all integrated cells. **(B)** Dot plot showing exclusively expressed marker genes (Pecam1 and Cdh5) in ECs across all the identified cell populations. **(C)** UMAP plot showing the distribution of ECs in sham group, 3, 7 days after MI. **(D)** Volcano plot showing differentially expressed genes of ECs at 7 days after MI compared with ECs at 3 days. **(E)** Hallmark GSEA analysis of differentially expressed genes 7 days after MI compared with cells at 3 days showing that ECs at 7 days were positively correlated with DNA packing and negatively correlated with myeloid leukocyte migration. **(F)** UMAP plot of T cells from integrated data in sham group, 3, 7 days after MI surgery. **(G)** T cells were repartitioned and renamed before further analysis and showed with the UMAP plot. **(H)** Dot plot with specific marker genes of T cell clusters showing heterogeneous expression of T cells. The dot size and scale color represented the percentage of expressed genes and the mean expression of each cell population, respectively. **(I,J)** GSEA of effector T cells with regulated T cells, indicating that effector T cells were positively correlated with cell activation and immune system processes **(I)** and negatively correlated with humoral immune responses and myeloid leukocyte differentiation **(J)**. **(K)** Single-cell regulatory network inference and clustering (SCENIC) analysis showing distinct regulons cross T cell clusters.

As shown in a previous analysis, ECs are placed at the center hub of cellular crosstalk by expressing abundant receptors during the healing process (Figure 2C). Hence, it is important to understand the functional changes of ECs after MI. We first identified an EC population in our integrated data with predominantly expressed EC markers, such as Pecam1 and Cdh5 (Figures 7A,B). Investigating the dynamics of endothelia, we found that ECs shrank at 3 days and further expanded at 7 days after MI (Figures 2B, 7C). Additionally, the ECs in the sham group and at 3 days were found to be unbiasedly clustered together compared with what has been found at 7 days, suggesting distinct gene programming of ECs at 7 days. Intriguingly, by analyzing differentially expressed genes, we found a distinct transcriptional profile of ECs at 7 days compared with what has been found at 3 days. Specifically, ECs at 3 days after surgery expressed higher levels of cytokines and chemokines, for instance, Ccl9, Cxcl2, which is consistent with the activated pathways involved in myeloid leukocyte migration (Figures 7D,E). Enriching of the profibrotic gene Col3a1, pro-proliferating gene Tpt1, and ribosomal proteins Rpl9 and Rps12 at 7 days, fitting its positively correlated pathway of DNA packing, indicated an active transition of proinflammatory to proreparative ECs from 3 to 7 days after MI (Figures 7D,E). Overall, these observations confirmed the differentially expressed profile of ECs during the healing process.

Notably, a growing body of literature has emphasized the crucial functions of lymphocytes, especially T cells, in cardiac remodeling after MI on the basis of their important role in initiating and regulating immune responses (36). To uncover the underlying subtypes of T cells, we collected T cell clusters for the next analysis. Dynamic analysis of T cells among sham, MI-3d, and MI-7d indicated the occurrence of significantly extravasated T cells at 7 days after MI (Figure 7F). To reveal their heterogenous response after MI stimulation, T cells were repartitioned into four clusters: naïve T cells, effector T cells, regulated T cells, and NK cells (Figure 7G). Cells belonging to the naïve T cell cluster specifically express “naïve” marker genes, such as Tcf7 and CCR7 (37). On the other hand, the effector T cell cluster is characterized by upregulated expression of Ccl6 and Il1b, in line with positively enriched pathways about cell activation and regulation of immune system process in GSEA (Figures 7H,I). Conversely, negatively correlated inflammatory pathways were observed in the regulated T cell cluster (Figure 7J), for example, humoral immune response and myeloid leukocyte differentiation, indicating that divergent T cell clusters assume distinct anti-inflammatory and proinflammatory functions after MI. Regarding the particular differences in T cell subclusters during the healing process, a deeper understanding of the transcriptional factors inducing the transition of these cells would be important for the intervention of inflammatory responses after MI. Here, we performed a SCENIC regulon analysis and observed unique transcriptional regulons among these T cell clusters. Specifically, the effector T cells showed activated proinflammatory regulons, such as Irf5 and Fosb, whereas significantly activated Sp1 regulons in regulated T cells and Eomes regulons in NK cells were observed (Figure 7K). In conclusion, we discovered time-dependent and cell-cluster-dependent transcriptional programming of ECs and T cells and revealed their heterogeneous responses during the healing.

## Discussion

In this study, we integrated three single-cell sequencing datasets at 0, 3, and 7 days after MI surgery to obtain a more comprehensive understanding of the complex dynamics and gene programming of non-CMs under physiological and ischemic conditions. We demonstrated that activated FBs (Fibro_Myo) secrete predominant ligands for cell communication, whereas ECs possess a large number of receptors. Among all ligands and receptor pairs, the integrin family (e.g., Itgb1, Itga1, and Itgav) was identified as a pivotal interactive regulator during cardiac remodeling. Additionally, distinct functions of blood-derived and TR macrophages exhibiting altered gene profiles, regulons, and pathways were documented, allowing deeper understanding of the molecular mechanism of macrophages with different origins in response to ischemic stimulation. In addition, we showed that activated FBs (myofibroblasts) are characterized by a predominant level of Postn and Cthrc1. We also identified Ddah1, which is exclusively expressed in myofibroblasts in this study and was further confirmed using bulk RNA-seq, western blot, and immunofluorescence *in vivo* under normal and ischemic conditions. Collectively, this study provides a promising method to investigate the dynamics and cell transcriptional signature of different cell types simultaneously by integrating multiple single-cell sequencing data.

Notably, MI leads to sudden death of CMs, accompanied with the extravasation of leukocytes and lymphocytes from the blood vessels for clearance of necrotic debris, followed by replenishing of reparative cells, such as myofibroblasts, ECs, and anti-inflammatory macrophages (M2 macrophages) (2, 38). It is very important to understand the mechanism by which these cells finely orchestrate these proinflammatory and proreparative processes during cardiac remodeling via ligand and receptor interactions. In line with a previous study (10), the crosstalk among all subpopulations revealed that myofibroblasts and ECs are the key cell types involved in cellular communication, highlighting their pivotal role in remodeling processes. Moreover, the integrin family (e.g., Itgb1, Itga1, and Itgav) was found to be the most profound protein family participating in cellular crosstalk in our analysis.

Previous researches have confirmed that integrins contribute to myofibroblast differentiation by interacting with ECM or cytoskeleton proteins and activating the transcriptional expression of TGF-β in the heart, lungs, liver, and kidneys (39–42). Consistent with this effector, block or genetic-modulating their expression would attenuate the degree of fibrosis in these organs. Furthermore, a growing body of evidence has demonstrated that integrin-ECM interaction is indispensable for the growth, maturation, and integrity of great vessels during the development of the heart (43). In addition, under hypoxic stimulation, upregulated integrins promote angiogenesis by increasing EC migration and vascular-like tube formation (44). Notably, our results underscored the important role of myofibroblasts, endothelia, and integrins in cell-cell communication during cardiac remodeling after MI. However, the mechanism by which integrins participate in fibrosis and angiogenesis, which integrin are more weighted in this process, warrants deeper investigation in future studies.

Besides FBs, recent studies have emphasized the important role of immune cells, especially macrophages, in cardiac functional modulation under hemostatic and perturbed conditions (45). In this single-cell analysis, macrophages were found to constitute the largest proportion of immune cells in sham or post-ischemic hearts, highlighting their pivotal role in immune responses and cardiac remodeling. Emerging literatures have described the heterogenous effects of macrophages with different origins on immune responses and wound healing after MI, underscoring the urgent need to understand the function and transcriptional differences of TR and blood-derived macrophages (46). Notably, we partitioned all macrophages into seven subpopulations in this study. Incongruous expressed marker genes were observed among these clusters. Specifically, Cx3cr1 were found to be predominantly upregulated in TR macrophages, whereas Osm, which contribute to the production of cytokines, were found to be exclusively expressed in blood-derived macrophages. Differentially expressed Ccr2 has been previously described to be a hallmark for distinguishing TR and blood-derived macrophages using genetic lineage tracing (24). Macrophages with a low level of Ccr2 represent a TR population in human and mouse hearts and retain their quantity via self-proliferation, whereas macrophages with a high level of Ccr2 derived from monocytes are maintained via monocyte-dependent recruitment (47, 48). To determine the differences of transcriptional signature and pathways within these two cell clusters, by assigning all macrophages into Ccr2-high and Ccr2-low clusters, we discovered high levels of proreparative genes (e.g., Lyve1 and Mrc1) on Ccr2-low macrophages, consistent with the positively enriched pathways of cell proliferation and response to TGF-β. On the other hand, macrophages with a high level of Ccr2 were found to exhibit upregulated cytokines, such as Ccl2, Ccl7, and Ly6c2, matching the active pathways of exocytosis and myeloid cell activation. These findings indicate the divergent functions and gene signatures of these two cell types. Additionally, we explored the transcriptional regulons of all macrophage clusters, revealing several distinct transcription regulatory factors among TR and blood-derived macrophages. Specifically, Egr1 and Jund were found to be dramatically activated in Ccr2-low macrophages, whereas Ccr2-high macrophages were found to exhibit increased activity of Stat1 and Irf7. Intriguingly, evidence from several studies demonstrated that Egr1, the immediate early gene, participates in the differentiation of myeloid cell precursors along macrophage lineages (29). This phenomenon indicates the important role that Egr1 plays in the maintenance of TR macrophages. Notably, in this study, we not only confirmed the distinct gene profiles and pathways of TR and monocyte-derived macrophages in a single-cell resolution but also provided insights into their differentially activated transcript regulons that may contribute to these divergent functions. However, how TR and blood-derived macrophages orchestrate these complex proinflammatory and anti-inflammatory effects in a fine sequential manner under ischemic conditions and which regulons account for this transition remain elusive and need to be urgently explored in the future.

To our knowledge, this is the first study raising the question of whether it is effective to have a comprehensive understanding of non-CM flux as well as cellular crosstalk and functions under homeostatic and ischemic conditions by integrating multiple single-cell datasets. We believe that single-cell sequencing analysis of multiple cell populations in a cell-type- and disease-dependent manner would provide new opportunities for discovering novel drugs and intervention targets for the repair processes of the heart.

## Data Availability Statement

The original contributions presented in the study are included in the article/Supplementary Material, further inquiries can be directed to the corresponding author/s.

## Ethics Statement

The animal study was reviewed and approved by the Animal Care Committee of Shanghai Jiao Tong University School of Medicine.

## Author Contributions

LZ performed the study, analyzed the data, and wrote the manuscript. LL and RZ contributed to manuscript preparation and revision. KC and XY participated in the experimental design, data interpretation, manuscript preparation, and revision. All authors contributed to the article and approved the submitted version.

## Conflict of Interest

The authors declare that the research was conducted in the absence of any commercial or financial relationships that could be construed as a potential conflict of interest.

## Abbreviations

MI: myocardial infarction
scRNA-seq: single-cell RNA sequencing
non-CM: non-cardiomyocyte
SMC: smooth muscle cell
GSEA: gene set enrichment analysis
SCENIC: single-cell regulatory network inference and clustering
FB: fibroblast
EC: endothelial cell
TR: tissue resident
ECM: extracellular matrix.
