# Supplementary material for: Comprehensive Integration of Single-Cell Transcriptional Profiling Reveals the Heterogeneities of Non-cardiomyocytes in Healthy and Ischemic Hearts
Source: Front Cardiovasc Med. 2020 Dec 7;7:615161. doi: 10.3389/fcvm.2020.615161 (PMC7750309; doi:10.3389/fcvm.2020.615161)
Supplement: Supplementary file 1 [file Data_Sheet_1.DOCX]

**Supplementary Material**

**
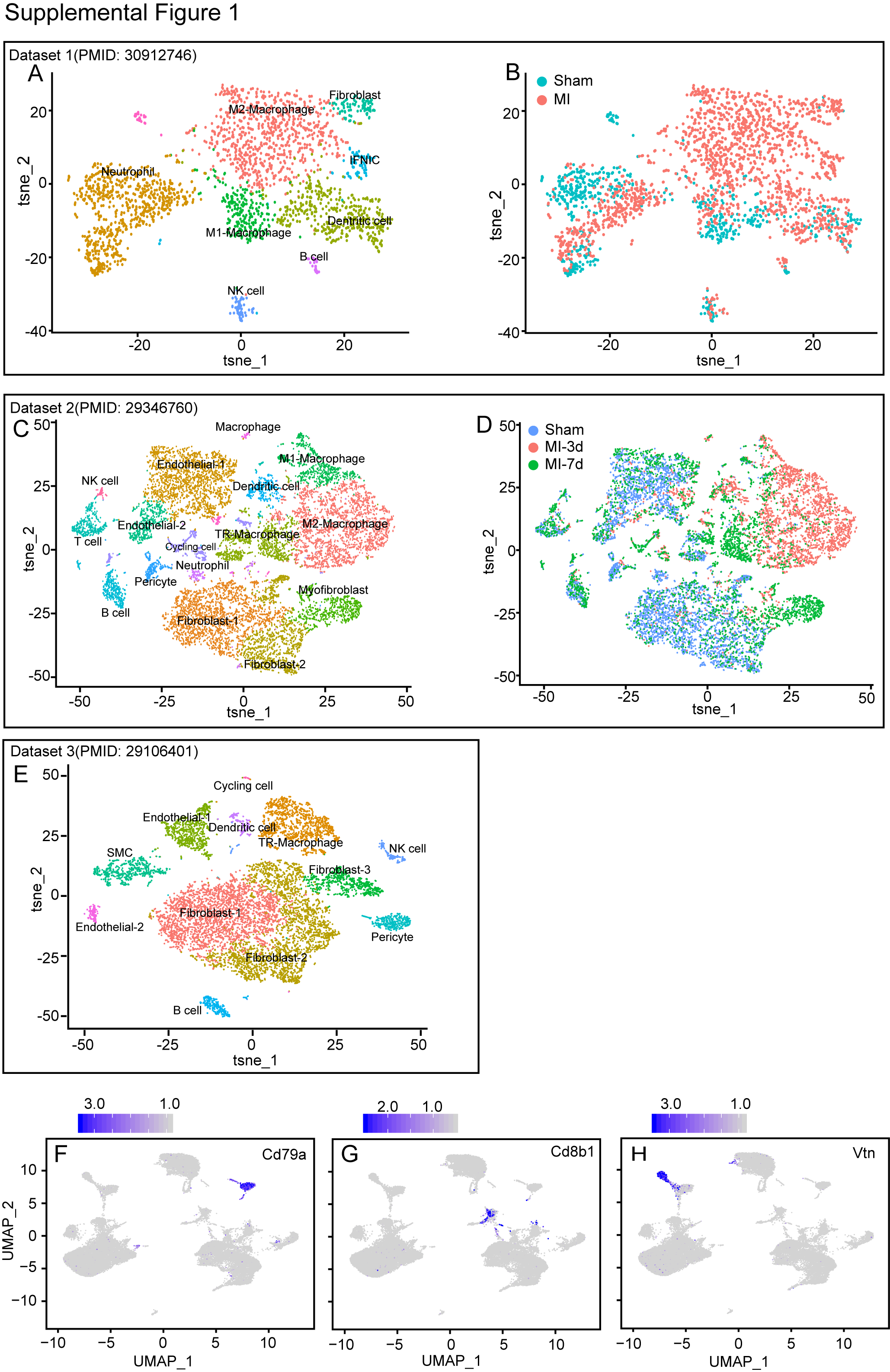
**

**Supplemental Figure 1. Integrating three datasets of non-cardiomyocytes (non-CMs) for a deeper analysis.** (A, B) Single cell-RNA sequencing dataset (PMID: 30912746) of all CD45+ cells from sham- or myocardial infarction-operated hearts (4 days). (C, D) Secondary single cell RNA sequencing dataset (PMID: 29346760) of all non-CMs from the sham group or 3 days, 7 days after MI surgery. (E) Third single cell RNA-sequencing dataset (PMID: 29106401) of non-CMs from healthy hearts. (F-H) UMAP plot including all cells showing the expression of Cd79a (F), Cd8a1 (G), and Vtn (H). The expression levels are indicated by scales in the upper portion of each panel.

**
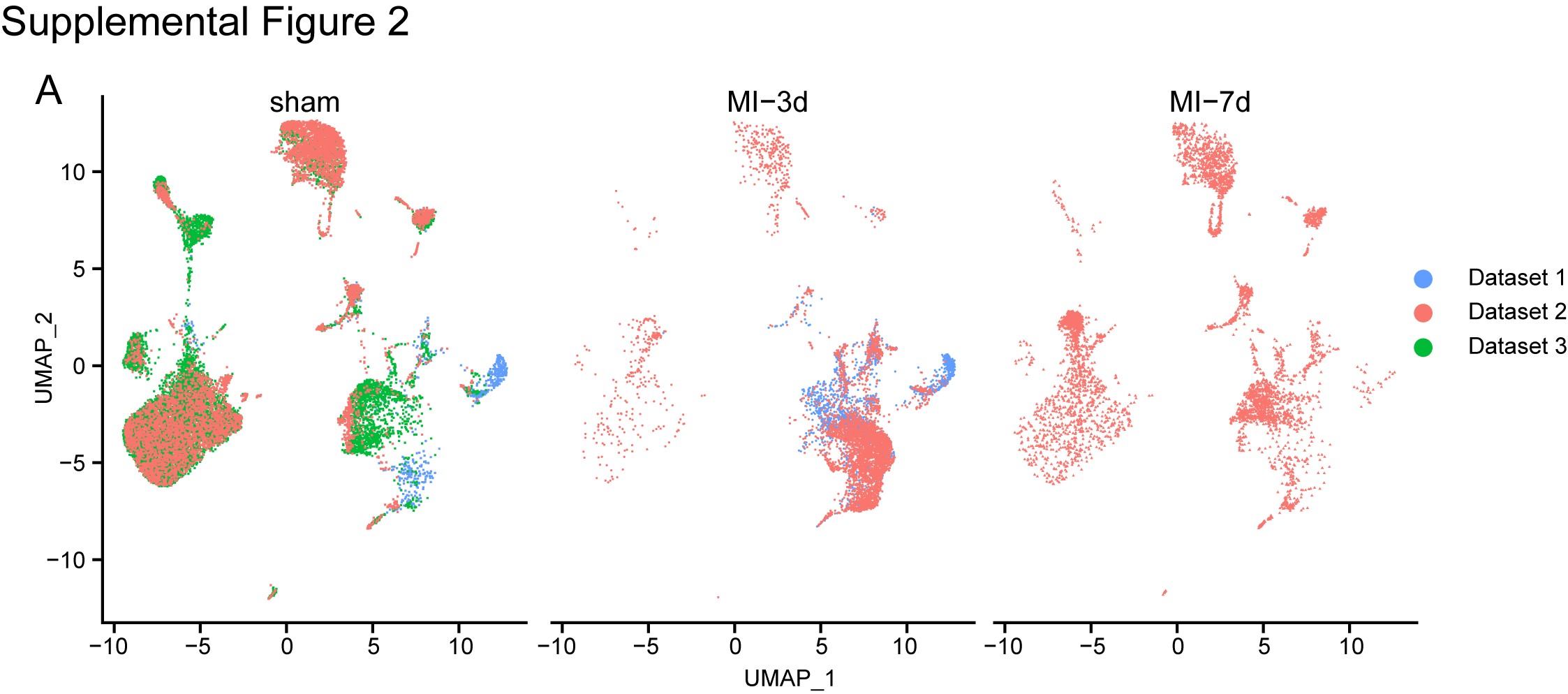
**

**Supplemental Figure 2. Three sources data of non-CMs in sham, 3-days, 7-days post-MI surgery.** (A) UMAP plot showed the source data of non-CMs from dataset1 (PMID: 30912746), dataset 2 (PMID: 29346760) and dataset 3 (PMID: 29106401) at sham, 3-days, 7-days post-MIsurgery.

**
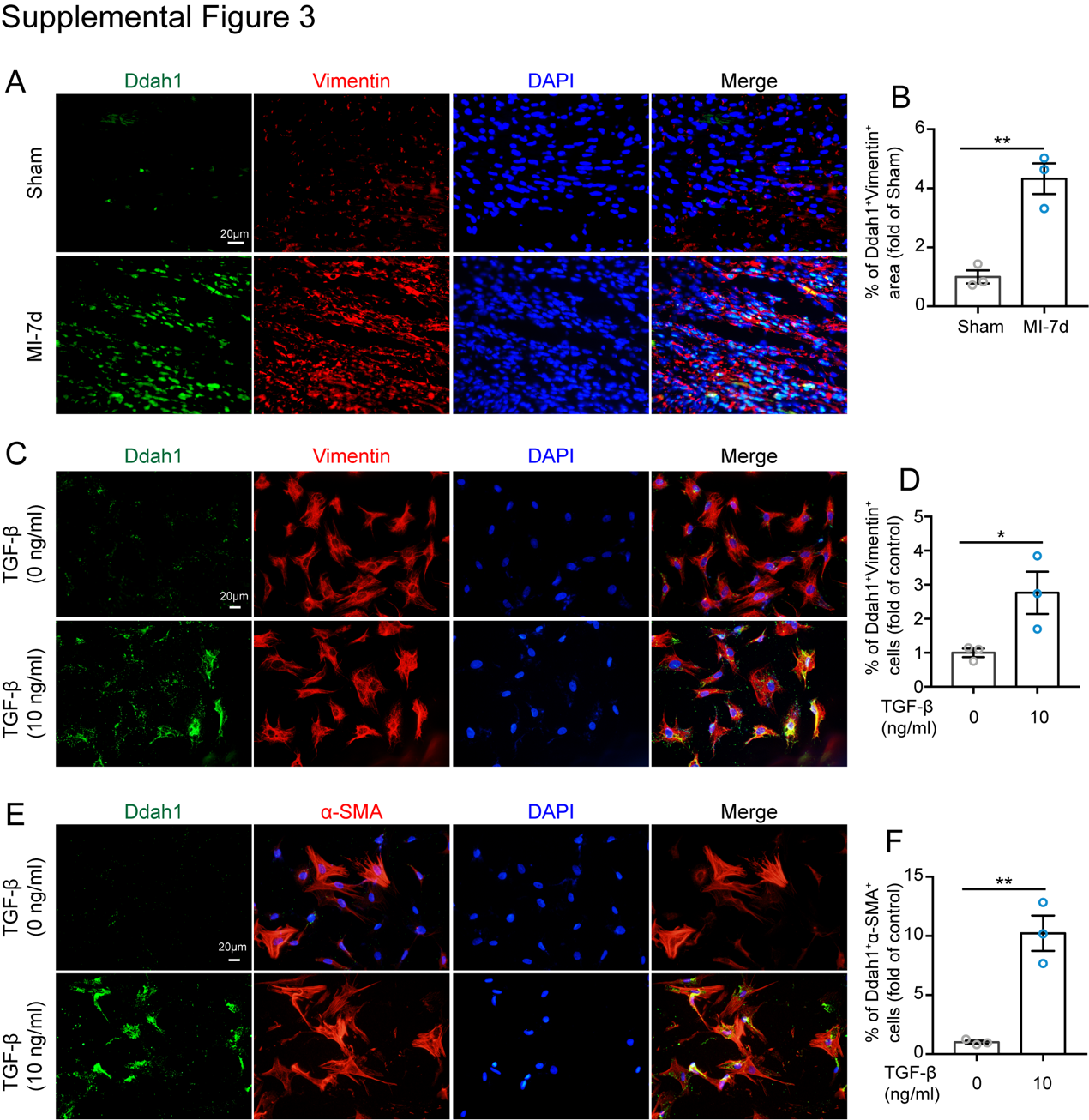
**

**Supplemental Figure 3. Upregulation of Ddah1 in fibroblasts after myocardial infarction (MI) or TGF-β treatment.** (A) Representative immunofluorescent images from three separate experiments showed co-expression of Ddah1 (green) and vimentin (red), nuclei were shown with DAPI (blue). (B) The quantification of panel A. (C, E) Fibroblasts were isolated from neonatal rat hearts and stained with Ddah1 (green) and vimentin (red, C) or ⍺-smooth muscle actin (α-SMA, red, E) after TGF-β treatment. (D) Quantification results in panel C. (F) Quantification results in panel E. ^*^*p* < 0.05, ^**^*p* < 0.01. [(B, D, F), *n* = 3, Student's *t* test.]

**
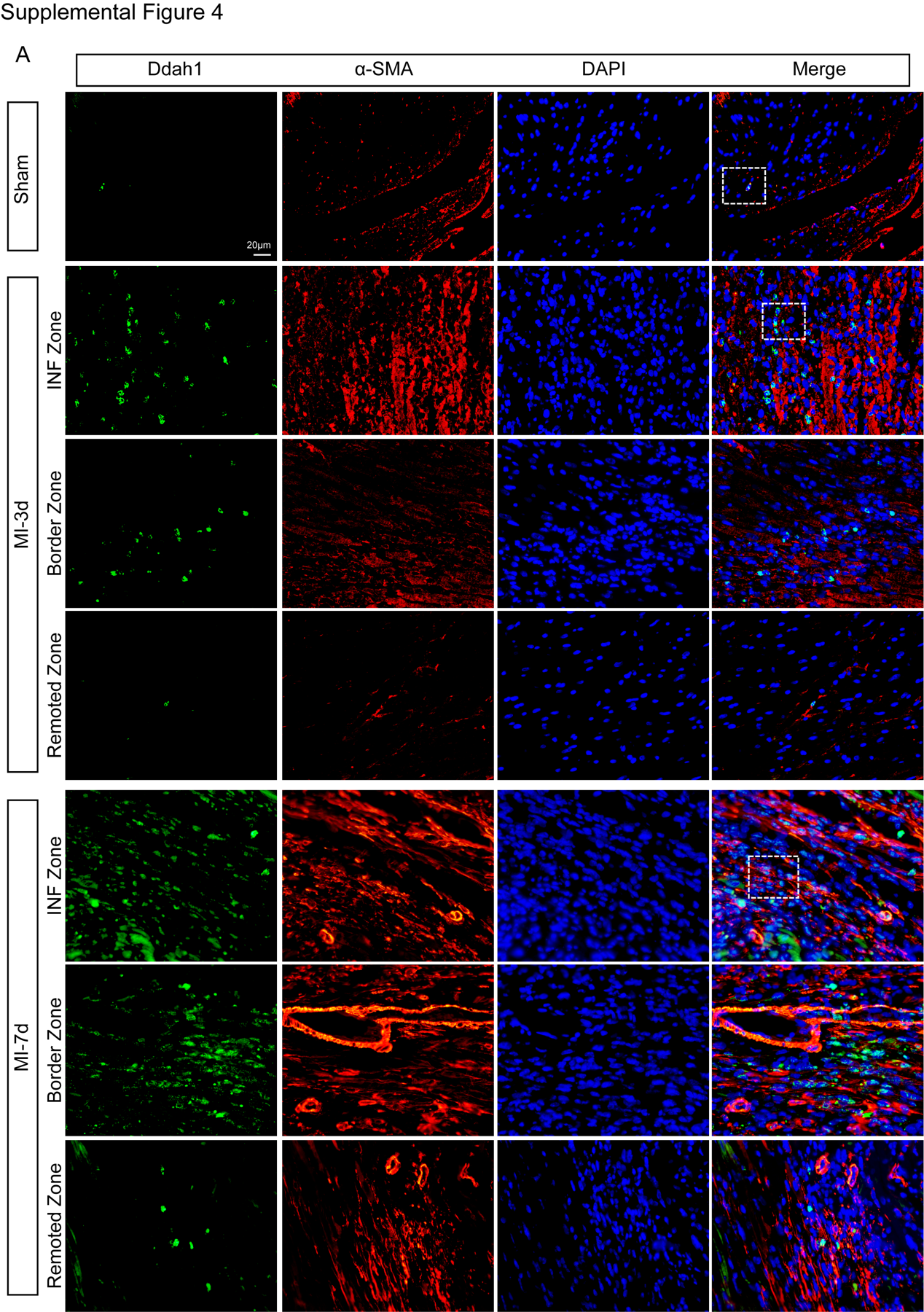
**

**Supplemental Figure 4. Upregulation of Ddah1 in myofibroblasts at 3-days and 7-days after myocardial infarction (MI).** (A) Representative immunofluorescent images showed the expression of Ddah1 (green) and α-SMA (red) in the infarcted (INF, corresponding to Figure 6E), border, and remoted regions at 3 days and 7 days after MI surgery or sham hearts. Nuclei were stained with DAPI.

**
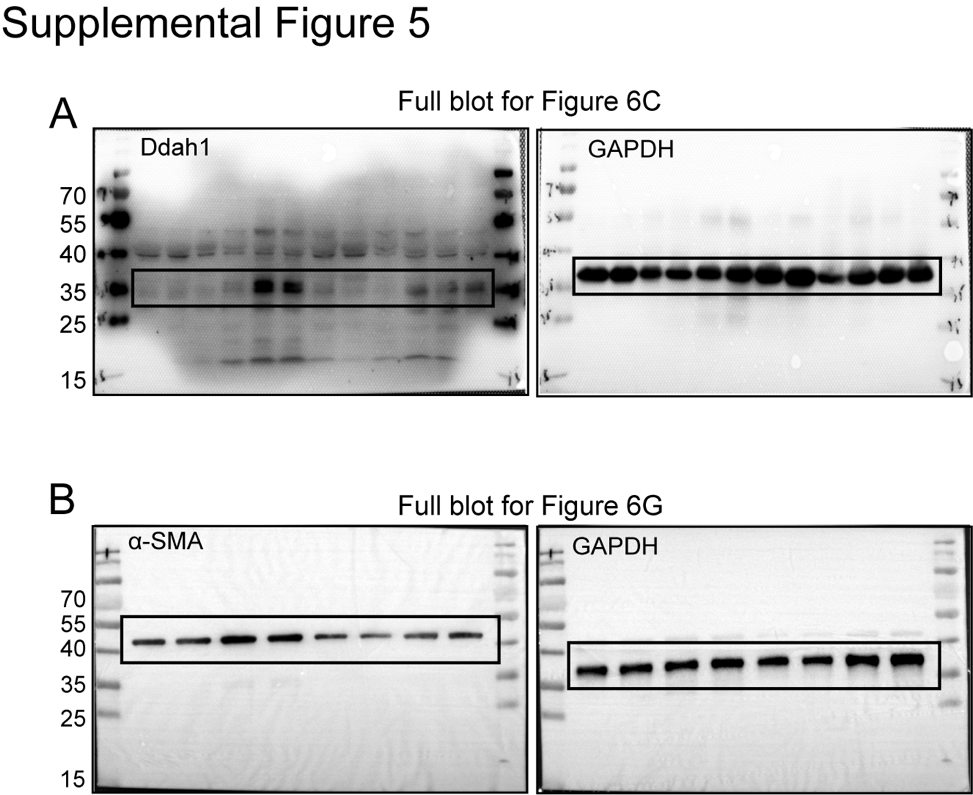
**

**Supplemental Figure 5. Unedited full blot images in this article.** (A) The full uncut gel corresponding to Figure 6C. Rectangle indicated target proteins. (B) The full uncut gel corresponding to Figure 6G. Rectangle indicated target proteins.

**List of Supplemental Tables**

**Supplemental Table I. Summary of scRNA-seq studies of hearts.**

**Supplemental Table II. Perfect genes for identifying all subpopulations.**

**Supplemental Table III. Summarized paths of cell communications in all subclusters.**

**Supplemental Table IV. Results of GSEA analysis between tissue resident (TR)- and blood born-macrophages.**

| **Supplemental Table I**  **Summary of scRNA-seq studies of hearts** | | | |
| --- | --- | --- | --- |
| **Publications** | **Design** | **Accession codes** | **Seq-Method** |
| Nona Farbehi, et al.(2019)(1)  PMID: 30912746 | Including **all interstitial cells** (e.g. fibroblasts, vascular and immune cells) from murine hearts at 3 or 7 days after sham or MI surgery. | E-MTAB-7376, E-MTAB-7365 | 10x Genomics Chromium |
| Ruiz Adrian, et al.(2019)(2) | Isolating **fibroblasts** from Col1a1-GFP reporter mice at 7, 14, 30 days after MI surgery and healthy hearts. |  |  |
| Monika M Gladka, et al.(2018)(3)  PMID: 29386203 | Including **all cardiac cell types** from infarcted areas or healthy mice hearts at 3 days after IR or sham surgery. | Unloaded in public databases | SORT-seq |
| Peng Hu, et al.(2018)(4)  PMID: 30254108 | Single-nucleus RNA sequencing of healthy hearts at 6 or 10 days postnatal mice or 10 days of ERRα/γ-KO hearts. | GSE118545 | sNucDrop-seq |
| Ziwen Li, et al.(2019)(5)  PMID: 31162546 | Including **vascular endothelial cells** from healthy or ischemic hearts at 7 days post MI surgery. | Unloaded in public databases | 10x Genomics Chromium |
| Daniel A Skelly, et al.(2018)(6)  PMID: 29346760 | Isolating nucleated **non-cardiomyocytes** from heart ventricles of female and male mice. | E-MTAB-6173 | 10x Genomics Chromium |
| Seitaro Nomura, et al.(2018)(7)  PMID: 30375404 | Isolating **cardiomyocytes** from TAC-operated hearts at 3 days, 1, 2, 4, 8 weeks or sham surgery. | GSE95143 | SMART-seq |
| Daniel M DeLaughter, et al.(2016)(8)  PMID: 27840107 | Collecting **all cardiac cell types** from embryonic hearts at day 9.5, 11.5, 14.5 and posnatal hearts at day 0, 21. | 272R, 274R, 275-292R, 439R, and 440R | SMART-seq |
| Sebastian Schafer, et al.(2017)(9)  PMID: 29160304 | Isolating **non-cardiomyocytes** from 18-week-old PLNR9C transgenic mice or its WT littermates hearts. | GSE97117 | 10x Genomics Chromium |
| Kevin R King, et al.(2017)(10)  PMID: 29106401 | Including CD45+ **leukocytes** from WT or Irf3-KO hearts at 4 days after MI or sham surgery. | GSE106473 | Drop-seq |
| Yang Xiao, et al.(2019)(11)  PMID: 31558567 | **Non-cardiomyocytes** from WT and Lats1/2 CKO hearts at 1 week post-MI operation. | GSE135296 | Drop-seq |
| Sarah A Dick, et al.(2019)(12)  PMID: 30538339 | Pooling cardiac **macrophages** and **dendritic cells** from WT mice; isolating macrophages from infarcted Cx3cr1CreER–YFP:R26Td and control hearts at days 11 after MI surgery. | GSE119355 | 10x Genomics Chromium |
| scRNA-seq, single cell-RNA sequencing; MI, myocardial infarction; IR, ischemia reperfusion; TAC, transverse aorta constriction; WT, wild type; KO, knock out.  Three datasets chosen for next analysis were highlighted in yellow. | | | |

| **Supplemental Table II**  **Perfect genes for identifying all subpopulations** | | | | | | |  |
| --- | --- | --- | --- | --- | --- | --- | --- |
| **Cell Identity** | **Gene** | **Avg_logFC** | **Pct.1** | **Pct.2** | **p_val** | **p_val_adj** | |
| Fibro_1 | Smoc2 | 1.82618005 | 0.977 | 0.422 | 0 | 0 | |
|  | Dpep1 | 1.7800272 | 0.96 | 0.376 | 0 | 0 | |
|  | Gpx3 | 1.73234808 | 0.987 | 0.51 | 0 | 0 | |
|  | Dcn | 1.68719729 | 1 | 0.74 | 0 | 0 | |
|  | Gsn | 1.66192513 | 1 | 0.86 | 0 | 0 | |
|  | Crispld2 | 1.64572492 | 0.965 | 0.388 | 0 | 0 | |
|  | Hsd11b1 | 1.64113645 | 0.9 | 0.355 | 0 | 0 | |
|  | Htra3 | 1.57572424 | 0.988 | 0.513 | 0 | 0 | |
|  | Serping1 | 1.45663639 | 0.988 | 0.517 | 0 | 0 | |
|  | Cxcl14 | 1.59877559 | 0.551 | 0.257 | 4.71E-288 | 9.42E-285 | |
| Endo-1 | Fabp4 | 3.18016824 | 0.997 | 0.819 | 0 | 0 | |
|  | Ly6c1 | 2.63095052 | 0.979 | 0.622 | 0 | 0 | |
|  | Cd36 | 2.47021758 | 0.968 | 0.529 | 0 | 0 | |
|  | Mgll | 2.31898287 | 0.926 | 0.557 | 0 | 0 | |
|  | Cav1 | 2.29867197 | 0.972 | 0.467 | 0 | 0 | |
|  | Cxcl12 | 2.20140656 | 0.866 | 0.5 | 0 | 0 | |
|  | Tspan13 | 1.94286908 | 0.937 | 0.551 | 0 | 0 | |
|  | Slc9a3r2 | 1.90305571 | 0.914 | 0.545 | 0 | 0 | |
|  | Icam2 | 1.89197085 | 0.886 | 0.532 | 0 | 0 | |
|  | Cd300lg | 1.85214318 | 0.788 | 0.472 | 0 | 0 | |
| MAC_M2 | Spp1 | 3.03537074 | 0.916 | 0.538 | 0 | 0 | |
|  | Arg1 | 2.39436838 | 0.69 | 0.402 | 0 | 0 | |
|  | Hmox1 | 2.07035162 | 0.784 | 0.347 | 0 | 0 | |
|  | Pf4 | 2.065784 | 0.771 | 0.367 | 0 | 0 | |
|  | Ccl2 | 2.03741114 | 0.852 | 0.42 | 0 | 0 | |
|  | Ccl7 | 2.01260249 | 0.738 | 0.346 | 0 | 0 | |
|  | Ccl6 | 1.94373736 | 0.949 | 0.341 | 0 | 0 | |
|  | Ccl9 | 1.93480153 | 0.944 | 0.328 | 0 | 0 | |
|  | Lgals3 | 1.86969818 | 0.987 | 0.455 | 0 | 0 | |
|  | Lgmn | 1.80407669 | 0.976 | 0.505 | 0 | 0 | |
| MAC-TR | C1qa | 2.38668811 | 0.979 | 0.42 | 0 | 0 | |
|  | C1qc | 2.24073871 | 0.975 | 0.367 | 0 | 0 | |
|  | C1qb | 2.22639186 | 0.981 | 0.452 | 0 | 0 | |
|  | Apoe | 1.881245 | 0.999 | 0.932 | 0 | 0 | |
|  | Ms4a7 | 1.76076813 | 0.914 | 0.356 | 0 | 0 | |
|  | Cd74 | 1.70502338 | 0.908 | 0.618 | 0 | 0 | |
|  | Fcrls | 1.54165291 | 0.816 | 0.201 | 0 | 0 | |
|  | Lyz2 | 1.40402366 | 1 | 0.758 | 0 | 0 | |
|  | Wfdc17 | 1.36229409 | 0.968 | 0.399 | 0 | 0 | |
|  | Saa3 | 1.44512259 | 0.605 | 0.367 | 1.05E-183 | 2.09E-180 | |
| Fibro_Myo | Postn | 2.49237014 | 0.935 | 0.419 | 0 | 0 | |
|  | Col3a1 | 1.90679144 | 0.996 | 0.654 | 0 | 0 | |
|  | Col1a1 | 1.82969742 | 0.994 | 0.55 | 0 | 0 | |
|  | Col8a1 | 1.80693866 | 0.972 | 0.515 | 0 | 0 | |
|  | Col1a2 | 1.7333822 | 0.995 | 0.599 | 0 | 0 | |
|  | Mfap4 | 1.72581283 | 0.909 | 0.389 | 0 | 0 | |
|  | Mfap5 | 1.62475536 | 0.99 | 0.573 | 0 | 0 | |
|  | Cthrc1 | 1.61159119 | 0.681 | 0.433 | 0 | 0 | |
|  | Timp1 | 1.52538318 | 0.88 | 0.479 | 0 | 0 | |
|  | Fstl1 | 1.50351523 | 0.997 | 0.559 | 0 | 0 | |
| Fibro_2 | Pi16 | 1.90310596 | 0.993 | 0.591 | 0 | 0 | |
|  | Cd248 | 1.38835213 | 0.86 | 0.271 | 0 | 0 | |
|  | Mfap5 | 1.36121229 | 0.998 | 0.581 | 0 | 0 | |
|  | Igfbp6 | 1.35907693 | 0.965 | 0.519 | 0 | 0 | |
|  | Pcolce2 | 1.28178999 | 0.983 | 0.444 | 0 | 0 | |
|  | Axl | 1.20789471 | 0.981 | 0.543 | 0 | 0 | |
|  | Dpt | 1.15393195 | 0.99 | 0.547 | 0 | 0 | |
|  | Cd34 | 1.12128114 | 0.995 | 0.68 | 0 | 0 | |
|  | Fbln2 | 1.0864807 | 0.991 | 0.579 | 0 | 0 | |
|  | Fbn1 | 1.08019508 | 0.936 | 0.503 | 0 | 0 | |
| Neutrophil | Retnlg | 2.56702812 | 0.859 | 0.319 | 0 | 0 | |
|  | Il1b | 2.56416161 | 0.968 | 0.344 | 0 | 0 | |
|  | Cxcl2 | 2.50515458 | 0.881 | 0.334 | 0 | 0 | |
|  | Slpi | 2.36660917 | 0.91 | 0.345 | 0 | 0 | |
|  | S100a9 | 4.28782802 | 0.825 | 0.544 | 5.21E-259 | 1.04E-255 | |
|  | S100a8 | 4.43333453 | 0.856 | 0.619 | 1.41E-250 | 2.83E-247 | |
|  | Marcksl1 | 2.05981187 | 0.752 | 0.436 | 3.79E-116 | 7.57E-113 | |
|  | Clec4e | 2.16109934 | 0.67 | 0.292 | 4.77E-98 | 9.54E-95 | |
|  | G0s2 | 2.09452674 | 0.845 | 0.509 | 6.30E-69 | 1.26E-65 | |
|  | Cxcr2 | 2.0665198 | 0.566 | 0.14 | 4.59E-34 | 9.19E-31 | |
| T/NK cell | Ms4a4b | 2.61391941 | 0.902 | 0.341 | 0 | 0 | |
|  | Cd3g | 2.02731513 | 0.85 | 0.29 | 0 | 0 | |
|  | Cd3d | 1.97110082 | 0.863 | 0.322 | 0 | 0 | |
|  | Ptprcap | 1.80100602 | 0.85 | 0.365 | 0 | 0 | |
|  | Hcst | 1.66217945 | 0.836 | 0.411 | 0 | 0 | |
|  | Lck | 1.63923711 | 0.793 | 0.254 | 0 | 0 | |
|  | Ltb | 1.64853788 | 0.808 | 0.378 | 9.36E-299 | 1.87E-295 | |
|  | Nkg7 | 2.38461498 | 0.699 | 0.277 | 3.28E-281 | 6.56E-278 | |
|  | Cd8b1 | 1.77095728 | 0.608 | 0.359 | 3.48E-153 | 6.97E-150 | |
|  | Ccl5 | 3.44622076 | 0.411 | 0.269 | 8.01E-49 | 1.60E-45 | |
| DC cell | Cd74 | 2.30857228 | 0.992 | 0.634 | 0 | 0 | |
|  | Napsa | 1.75596996 | 0.913 | 0.425 | 0 | 0 | |
|  | Klrd1 | 1.71104997 | 0.781 | 0.306 | 0 | 0 | |
|  | Cd209a | 2.0185009 | 0.686 | 0.228 | 1.89E-258 | 3.77E-255 | |
|  | H2afz | 1.34887397 | 0.986 | 0.846 | 2.92E-204 | 5.84E-201 | |
|  | Tnip3 | 1.35545961 | 0.669 | 0.326 | 1.01E-186 | 2.01E-183 | |
|  | Plbd1 | 1.38424022 | 0.77 | 0.35 | 1.98E-167 | 3.96E-164 | |
|  | Cd7 | 1.41624089 | 0.605 | 0.242 | 7.88E-165 | 1.58E-161 | |
|  | Ifitm1 | 1.84522671 | 0.648 | 0.3 | 7.30E-104 | 1.46E-100 | |
|  | H2afy | 1.33033252 | 0.828 | 0.697 | 1.94E-101 | 3.88E-98 | |
| MAC_Mo/M1 | Chil3 | 2.6595517 | 0.918 | 0.519 | 0 | 0 | |
|  | Plac8 | 2.42728129 | 0.989 | 0.503 | 0 | 0 | |
|  | Hp | 2.2423308 | 0.978 | 0.442 | 0 | 0 | |
|  | Lyz2 | 2.01417715 | 1 | 0.774 | 0 | 0 | |
|  | Ifitm6 | 1.96724262 | 0.907 | 0.393 | 0 | 0 | |
|  | Gsr | 1.59908567 | 0.919 | 0.513 | 0 | 0 | |
|  | S100a4 | 1.53651212 | 0.974 | 0.596 | 0 | 0 | |
|  | Sirpb1c | 1.56870195 | 0.885 | 0.47 | 8.58E-297 | 1.72E-293 | |
|  | Ly6c2 | 1.91407584 | 0.846 | 0.423 | 8.87E-292 | 1.77E-288 | |
|  | Napsa | 1.56258251 | 0.867 | 0.427 | 1.76E-284 | 3.53E-281 | |
| B cell | Cd79a | 3.1105055 | 0.993 | 0.324 | 0 | 0 | |
|  | Cd79b | 2.86727331 | 0.994 | 0.424 | 0 | 0 | |
|  | Ly6d | 2.86198608 | 0.959 | 0.316 | 0 | 0 | |
|  | Ms4a1 | 2.38046633 | 0.941 | 0.359 | 0 | 0 | |
|  | Ltb | 2.16781907 | 0.915 | 0.377 | 0 | 0 | |
|  | Ptprcap | 2.02959526 | 0.936 | 0.365 | 0 | 0 | |
|  | Cd37 | 1.99420163 | 0.923 | 0.328 | 0 | 0 | |
|  | Mzb1 | 1.95908085 | 0.836 | 0.29 | 0 | 0 | |
|  | Fcmr | 1.92488383 | 0.849 | 0.37 | 0 | 0 | |
|  | Ccr7 | 2.11678882 | 0.8 | 0.391 | 5.19E-267 | 1.04E-263 | |
| SMC | Acta2 | 3.6218497 | 0.992 | 0.318 | 0 | 0 | |
|  | Tagln | 2.9295977 | 0.982 | 0.191 | 0 | 0 | |
|  | Myl9 | 2.72224093 | 0.992 | 0.28 | 0 | 0 | |
|  | Tpm2 | 2.37596568 | 0.989 | 0.341 | 0 | 0 | |
|  | Tpm1 | 1.97646986 | 0.992 | 0.587 | 0 | 0 | |
|  | Ndufa4l2 | 1.70663901 | 0.987 | 0.442 | 0 | 0 | |
|  | Cald1 | 1.64647626 | 0.993 | 0.674 | 0 | 0 | |
|  | Pdgfrb | 1.12570072 | 0.98 | 0.379 | 9.98E-308 | 2.00E-304 | |
|  | Des | 1.58802554 | 0.878 | 0.192 | 1.88E-304 | 3.77E-301 | |
|  | Nr2f2 | 1.09992708 | 0.768 | 0.258 | 6.92E-146 | 1.38E-142 | |
| Fibro_3 | Mgp | 1.90193464 | 1 | 0.808 | 0 | 0 | |
|  | Ccdc80 | 1.8417972 | 0.997 | 0.626 | 0 | 0 | |
|  | Fbln1 | 1.40493245 | 0.993 | 0.515 | 0 | 0 | |
|  | Serpinf1 | 1.26821842 | 0.993 | 0.558 | 5.21E-275 | 1.04E-271 | |
|  | Timp3 | 1.34627036 | 0.976 | 0.6 | 3.64E-260 | 7.29E-257 | |
|  | Dcn | 1.24562983 | 0.999 | 0.793 | 2.87E-256 | 5.74E-253 | |
|  | Lmo4 | 1.25843358 | 0.967 | 0.559 | 3.22E-254 | 6.43E-251 | |
|  | Cfh | 1.26079375 | 0.98 | 0.601 | 6.38E-252 | 1.28E-248 | |
|  | Mfap4 | 1.79268179 | 0.911 | 0.421 | 4.20E-238 | 8.41E-235 | |
|  | Prg4 | 1.9113258 | 0.769 | 0.255 | 8.83E-186 | 1.77E-182 | |
| Pericyte | Steap4 | 2.66783763 | 0.967 | 0.208 | 0 | 0 | |
|  | Ndufa4l2 | 2.48096808 | 0.989 | 0.445 | 0 | 0 | |
|  | Pdgfrb | 2.44971719 | 0.987 | 0.382 | 0 | 0 | |
|  | Mfge8 | 2.07050259 | 0.991 | 0.586 | 0 | 0 | |
|  | Gng11 | 1.5628382 | 0.998 | 0.713 | 5.55E-267 | 1.11E-263 | |
|  | Rasgrp2 | 1.67046091 | 0.942 | 0.463 | 7.50E-247 | 1.50E-243 | |
|  | Plxdc1 | 1.43432705 | 0.81 | 0.301 | 2.10E-214 | 4.19E-211 | |
|  | Cald1 | 1.42479941 | 0.986 | 0.676 | 2.37E-209 | 4.74E-206 | |
|  | P2ry14 | 1.7682368 | 0.777 | 0.289 | 2.26E-176 | 4.52E-173 | |
|  | Tpm2 | 1.41103598 | 0.819 | 0.348 | 4.71E-169 | 9.42E-166 | |
| MAC_3 | Stmn1 | 2.05407495 | 0.948 | 0.369 | 4.37E-194 | 8.75E-191 | |
|  | H2afz | 1.52600772 | 1 | 0.848 | 2.18E-151 | 4.36E-148 | |
|  | Top2a | 1.58502853 | 0.774 | 0.285 | 8.19E-140 | 1.64E-136 | |
|  | Hmgb2 | 1.60721813 | 0.967 | 0.575 | 8.62E-140 | 1.72E-136 | |
|  | Ube2c | 1.22784225 | 0.684 | 0.192 | 8.18E-131 | 1.64E-127 | |
|  | C1qb | 1.47674216 | 0.913 | 0.494 | 1.02E-119 | 2.05E-116 | |
|  | C1qa | 1.41194959 | 0.916 | 0.464 | 1.86E-116 | 3.71E-113 | |
|  | Birc5 | 1.50590557 | 0.733 | 0.303 | 4.44E-116 | 8.89E-113 | |
|  | C1qc | 1.32943578 | 0.891 | 0.415 | 4.96E-113 | 9.93E-110 | |
|  | Tk1 | 1.26588047 | 0.629 | 0.263 | 3.58E-68 | 7.17E-65 | |
| MAC_APC | C1qc | 1.42660545 | 0.926 | 0.416 | 1.40E-107 | 2.80E-104 | |
|  | C1qa | 1.45537286 | 0.915 | 0.466 | 1.73E-101 | 3.46E-98 | |
|  | C1qb | 1.37029578 | 0.933 | 0.495 | 1.24E-100 | 2.48E-97 | |
|  | Apoe | 1.18801052 | 1 | 0.937 | 2.16E-85 | 4.33E-82 | |
|  | Cx3cr1 | 0.91882407 | 0.766 | 0.341 | 3.06E-80 | 6.12E-77 | |
|  | Ms4a7 | 1.10847674 | 0.83 | 0.401 | 8.92E-80 | 1.78E-76 | |
|  | Fcrls | 0.95809304 | 0.72 | 0.251 | 4.03E-76 | 8.06E-73 | |
|  | Cd72 | 0.78506737 | 0.652 | 0.363 | 1.08E-49 | 2.16E-46 | |
|  | Ccl4 | 0.84348153 | 0.613 | 0.295 | 1.20E-48 | 2.39E-45 | |
|  | Cd74 | 1.08551878 | 0.848 | 0.642 | 9.17E-46 | 1.83E-42 | |
| MAC_4 | Ear2 | 2.46528972 | 0.826 | 0.316 | 4.69E-125 | 9.38E-122 | |
|  | Gngt2 | 1.79404797 | 0.988 | 0.618 | 1.23E-123 | 2.46E-120 | |
|  | Ifitm6 | 1.77657833 | 0.887 | 0.402 | 3.62E-122 | 7.24E-119 | |
|  | Lst1 | 1.61853749 | 0.984 | 0.446 | 7.28E-121 | 1.46E-117 | |
|  | Msrb1 | 1.64928018 | 0.972 | 0.638 | 2.37E-115 | 4.73E-112 | |
|  | Napsa | 1.47752642 | 0.919 | 0.434 | 2.75E-110 | 5.50E-107 | |
|  | Plac8 | 1.87254957 | 0.927 | 0.512 | 5.55E-107 | 1.11E-103 | |
|  | Itgal | 1.77476544 | 0.826 | 0.344 | 8.25E-105 | 1.65E-101 | |
|  | Pou2f2 | 1.61386325 | 0.883 | 0.434 | 2.25E-101 | 4.51E-98 | |
|  | Pglyrp1 | 1.70546116 | 0.684 | 0.405 | 1.24E-51 | 2.49E-48 | |
| IFNIC | Pyhin1 | 2.22617036 | 0.955 | 0.363 | 2.10E-147 | 4.19E-144 | |
|  | Ms4a4c | 2.32344731 | 0.95 | 0.452 | 2.67E-130 | 5.34E-127 | |
|  | Isg15 | 2.385216 | 0.946 | 0.519 | 2.14E-122 | 4.27E-119 | |
|  | Ifit1 | 2.25419014 | 0.893 | 0.444 | 2.62E-116 | 5.24E-113 | |
|  | Ifit3 | 2.14992903 | 0.897 | 0.385 | 9.62E-116 | 1.92E-112 | |
|  | Rsad2 | 2.6308664 | 0.893 | 0.425 | 2.46E-114 | 4.93E-111 | |
|  | Irf7 | 2.10371699 | 0.897 | 0.475 | 8.88E-107 | 1.78E-103 | |
|  | Ccl12 | 2.25215587 | 0.781 | 0.293 | 9.93E-88 | 1.99E-84 | |
|  | Cxcl10 | 2.80180733 | 0.669 | 0.269 | 5.93E-67 | 1.19E-63 | |
|  | Plac8 | 2.04557765 | 0.826 | 0.513 | 1.67E-63 | 3.35E-60 | |
| Fibro_4 | Plp1 | 2.93380574 | 1 | 0.158 | 9.48E-144 | 1.90E-140 | |
|  | Prnp | 3.14305216 | 1 | 0.513 | 1.17E-120 | 2.34E-117 | |
|  | Cryab | 2.36022672 | 1 | 0.501 | 1.14E-113 | 2.29E-110 | |
|  | Dbi | 2.40758866 | 0.994 | 0.821 | 5.23E-109 | 1.05E-105 | |
|  | Fxyd1 | 1.99623273 | 1 | 0.523 | 5.63E-109 | 1.13E-105 | |
|  | Cnp | 2.23292685 | 0.966 | 0.439 | 1.27E-106 | 2.54E-103 | |
|  | Pdlim4 | 2.01167929 | 0.954 | 0.333 | 3.69E-103 | 7.39E-100 | |
|  | Prss23 | 1.89155497 | 0.989 | 0.481 | 8.14E-96 | 1.63E-92 | |
|  | Cd59a | 1.92296362 | 0.863 | 0.304 | 1.72E-71 | 3.43E-68 | |
|  | Gatm | 1.86088244 | 0.766 | 0.266 | 7.67E-44 | 1.53E-40 | |
| **Avg_logFC:** log fold-change of the average expression between the two groups.  **Pct.1:** The percentage of cells where the genes is detected in the first group.  **Pct.2:** The percentage of cells where the genes is detected in the second group.  **p_val:** p_value (unadjusted).  **p_val_adj:** Adjusted p-value, based on bonferroni correction using all features in the dataset. | | | | | | | |

**Supplemental References**

[1] N. Farbehi, R. Patrick, A. Dorison, M. Xaymardan, V. Janbandhu, K. Wystub-Lis, et al. (2019). Single-cell expression profiling reveals dynamic flux of cardiac stromal, vascular and immune cells in health and injury. Elife. 8. doi: 10.7554/eLife.43882.

[2] A. Ruiz-Villalba, J.P. Romero, S.C. Hernandez, A. Vilas-Zornoza, N. Fortelny, L. Castro-Labrador, et al. (2020). Single-Cell RNA-seq Analysis Reveals a Crucial Role for Collagen Triple Helix Repeat Containing 1 (CTHRC1) Cardiac Fibroblasts after Myocardial Infarction. Circulation. doi: 10.1161/circulationaha.119.044557.

[3] M.M. Gladka, B. Molenaar, H. de Ruiter, S. van der Elst, H. Tsui, D. Versteeg, et al. (2018). Single-Cell Sequencing of the Healthy and Diseased Heart Reveals Cytoskeleton-Associated Protein 4 as a New Modulator of Fibroblasts Activation. Circulation. 138: 166-180. doi: 10.1161/circulationaha.117.030742.

[4] P. Hu, J. Liu, J. Zhao, B.J. Wilkins, K. Lupino, H. Wu, et al. (2018). Single-nucleus transcriptomic survey of cell diversity and functional maturation in postnatal mammalian hearts. Genes Dev. 32: 1344-1357. doi: 10.1101/gad.316802.118.

[5] Z. Li, E.G. Solomonidis, M. Meloni, R.S. Taylor, R. Duffin, R. Dobie, et al. (2019). Single-cell transcriptome analyses reveal novel targets modulating cardiac neovascularization by resident endothelial cells following myocardial infarction. Eur Heart J. 40: 2507-2520. doi: 10.1093/eurheartj/ehz305.

[6] D.A. Skelly, G.T. Squiers, M.A. McLellan, M.T. Bolisetty, P. Robson, N.A. Rosenthal, et al. (2018). Single-Cell Transcriptional Profiling Reveals Cellular Diversity and Intercommunication in the Mouse Heart. Cell Rep. 22: 600-610. doi: 10.1016/j.celrep.2017.12.072.

[7] S. Nomura, M. Satoh, T. Fujita, T. Higo, T. Sumida, T. Ko, et al. (2018). Cardiomyocyte gene programs encoding morphological and functional signatures in cardiac hypertrophy and failure. Nat Commun. 9: 4435. doi: 10.1038/s41467-018-06639-7.

[8] D.M. DeLaughter, A.G. Bick, H. Wakimoto, D. McKean, J.M. Gorham, I.S. Kathiriya, et al. (2016). Single-Cell Resolution of Temporal Gene Expression during Heart Development. Dev Cell. 39: 480-490. doi: 10.1016/j.devcel.2016.10.001.

[9] S. Schafer, S. Viswanathan, A.A. Widjaja, W.W. Lim, A. Moreno-Moral, D.M. DeLaughter, et al. (2017). IL-11 is a crucial determinant of cardiovascular fibrosis. Nature. 552: 110-115. doi: 10.1038/nature24676.

[10] K.R. King, A.D. Aguirre, Y.X. Ye, Y. Sun, J.D. Roh, R.P. Ng, Jr., et al. (2017). IRF3 and type I interferons fuel a fatal response to myocardial infarction. Nat Med. 23: 1481-1487. doi: 10.1038/nm.4428.

[11] Y. Xiao, M.C. Hill, L. Li, V. Deshmukh, T.J. Martin, J. Wang, et al. (2019). Hippo pathway deletion in adult resting cardiac fibroblasts initiates a cell state transition with spontaneous and self-sustaining fibrosis. Genes Dev. 33: 1491-1505. doi: 10.1101/gad.329763.119.

[12] S.A. Dick, J.A. Macklin, S. Nejat, A. Momen, X. Clemente-Casares, M.G. Althagafi, et al. (2019). Self-renewing resident cardiac macrophages limit adverse remodeling following myocardial infarction. Nat Immunol. 20: 29-39. doi: 10.1038/s41590-018-0272-2.
